# Green-synthesized silver nanoparticle-modulated polydiacetylene-based colorimetric pesticide sensor†

**DOI:** 10.1039/d5ra01243k

**Published:** 2025-06-02

**Authors:** Shazidul Hussain, Dipan Sarma, Sangita Majumder, Debajyoti Bhattacharjee, Khuloud A. Alibrahim, Abdullah N. Alodhayb, Hemant Agarwal, Syed Arshad Hussain

**Affiliations:** a Thin Film and Nanoscience Laboratory, Department of Physics, Tripura University Suryamaninagar Agartala 799022 Tripura India sa_h153@hotmail.com sahussain@tripurauniv.ac.in +913812374802 +91940212250; b Department of Botany, Tripura University Suryamaninagar Agartala 799022 Tripura India; c Department of Chemistry, College of Science, Princess Nourah bint Abdulrahman University Riyadh 11671 Saudi Arabia; d Research Chair for Tribology, Surface, and Interface Sciences, Department of Physics and Astronomy, College of Science, King Saud University Riyadh Saudi Arabia; e Barcelona School of Telecommunications Engineering, Universitat Politècnica de Catalunya Barcelona Tech, Carrer de Jordi Girona, 1-3, Les Corts 08034 Barcelona Spain

## Abstract

Recently, pesticide contamination has become a major threat to public health and ecosystems owing to its widespread and uncontrolled use in agriculture. Herein, we introduce a paper-based colorimetric sensing platform using polydiacetylene (PDA) that can be used as an efficient and cost-effective method for detecting pesticide residues. The sensor is designed on a nitrate cellulose membrane using 10,12-henicosadiynoic acid (HCDA), monomer of PDA, green-synthesized silver nanoparticles (AgNPs) and saponite clay (SC). The AgNPs were synthesized from *Araucaria heterophylla* leaf extract following a green synthesis protocol. The synthesized AgNPs were characterized through UV-Vis absorption, FESEM, TEM, DLS, XRD and FTIR spectroscopy. Moreover, the PDA phase change in the presence of AgNPs was optimized by varying AgNP concentration. The designed paper sensors responded to three commonly used pesticides, namely, cypermethrin (P_1_), pretilachlor (P_2_) and chlorpyriphos/cypermethrin (P_3_). It was found that the detection of different pesticides in different concentration regions can be achieved by varying AgNP to HCDA ratios. To determine pesticide concentration from the colorimetric response, a MATLAB-based program was developed for analyzing the RGB values corresponding to colour changes before and after exposure. The proposed sensor is similar to litmus paper in which sensing is achieved based on the visible colour change upon pesticide exposure. Therefore, it is economical and easy to use. This pesticide-detecting sensor provides measurements with high accuracy in a wide range of ambient conditions and is therefore a perfect portable system for point-of-care/on-site detection of pesticide residues.

## Abstract 2

Recently, pesticide contamination has become a major threat to public health and ecosystems owing to its widespread and uncontrolled use in agriculture.

## Graphical abstract

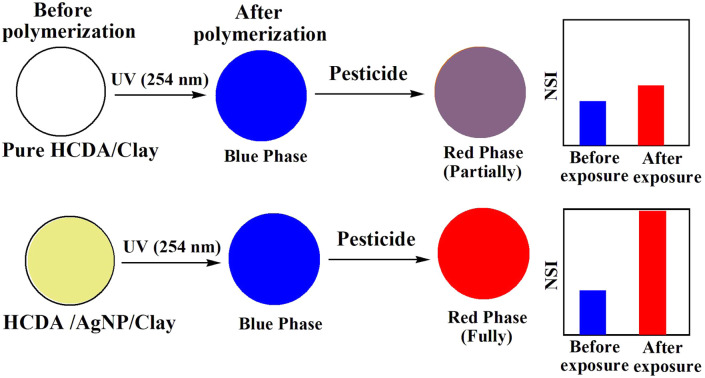

## 1 Introduction

The widespread and uncontrolled use of pesticides in agriculture to boost crop yield and enhance the quality of agricultural goods has become a serious threat to the environment in recent times. Excessive use of pesticides has led to major environmental and food pollution, making it one of the world's most concerning public health issues. It has been reported that around 300 000 people worldwide are dying from pesticide poisoning each year, and millions more were estimated to have been poisoned by this pesticide pollution.^1^ In addition to public health, pesticide pollution affects animals, aquatic species, and the environment. As a whole, the ecosystem gets disturbed. Therefore, the development of cost-effective and easy-to-use pesticide sensing tools is critical for ensuring sustainable agriculture to optimize the controlled use of pesticides with minimum environmental and health hazards. There exist different techniques such as enzyme-linked immunosorbent assay (ELISA),^2^ fluorescence-based assays,^3,4^ gas chromatography-mass spectrometry (GC-MS),^5^ liquid chromatography-mass spectrometry (LC-MS)^6^ and high-performance liquid chromatography (HPLC)^7^ for pesticide sensing. Despite the fact that these traditional techniques provide powerful trace analysis with good sensitivity and reproducibility, there are many drawbacks. Traditional methods require sophisticated, expensive instruments, sample preparation and purification steps, a longer time, professional technicians, and limited on-site and real-time applications, especially in emergency situations. Accordingly, the development of an innovative, cost-effective and eco-friendly pesticide sensing platform that is responsive to varying agricultural conditions worldwide is highly desirable. There exist very few reports wherein colorimetric pesticide detection systems have been proposed.^8–12^ In this work, the design of a PDA-based colorimetric paper senor for rapid detection of pesticides is explored. The currently used paper-based sensor is like litmus paper and very easy to use. Anyone can use this on the field *in situ*. Just by observing the colour change, it is possible to have an idea about the presence of pesticides. A detailed comparison study of already reported colorimetric PDA/NP-based sensors is summarized in Table 1. It has been observed that although there are several reports highlighting on the PDA/NP system for sensing applications, hardly there are any reports on the PDA/NP-based sensor. Only two PDA-based pesticide sensors have been reported.^11,12^ In both the cases, NPs were not involved. According to the literature survey, the present study is most probably the first attempt to design and study PDA/NP-based pesticide sensors with the demonstration of real-world sensing applications.

**Table 1:** Comparison of PDA/NP systems and selected pesticide sensors in terms of target analytes, detection limits, and selectivity

Sl no.	Materials	Target analytes	Limit of detection	Selectivity test	Reference
1	PDA & AgNP	N/A	N/A	N/A	27
2	PDA and silica NP	Cetyltrimethylammonium bromide	N/A	N/A	28
3	PDA and Au/Ag NPs	Organic solvents	N/A	Yes	29
4	PDA and AuNPs	N/A	N/A	N/A	30
5	PDA & zinc oxide NPs	Cetyltrimethylammonium bromide	N/A	N/A	31
6	PDA and AuNPs	Pb^2+^	N/A	Yes	32
7	PDA and AuNPs	Thrombin	N/A	N/A	33
8	PDA and magnetite (Fe_3_O_4_)	Sodium cetyltrimethylammonium bromide and streptavidin	30 μM	N/A	34
9	PDA and AuNPs	N/A	N/A	N/A	35
10	PDA and AgNPs	N/A	N/A	N/A	36
11	PDA and AuNPs	N/A	N/A	N/A	37
12	PDA and AuNPs	Human immunoglobulin E	0.1 ng mL^−1^	Yes	38
13	PDA and Fe_3_O_4_ NPs	Magnetic field	N/A	N/A	39
14	PDA/silica nanocomposite	Temperature	N/A	N/A	40
15	PDA and AgNPs	N/A	N/A	N/A	41
16	PDA and AgNPs	N/A	N/A	N/A	42
17	PDA and AgNPs	N/A	N/A	N/A	43
18	PDA and AgNPs	N/A	N/A	N/A	44
19	PDA and AgNPs	Temperature	N/A	N/A	26
20	PDA and AgNPs	N/A	N/A	N/A	45
21	PDA and AgNPs	N/A	N/A	N/A	46
22	AChE:AuNPs	Paraoxon (pesticide)	60 μM	Yes	8
23	AuNP	Malathion (pesticide)	139 mg L^−1^	Yes	9
24	Ag–Au	Malathion (pesticide)	1189 mg L^−1^	Yes	9
25	RB-AuNPs	Thiodicarb (pesticide)	0.08 mg L^−1^	Yes	10
26	PDA-PAM	Malathion (pesticide)	8 mM	Yes	11
27	PDA-HBA	Organophosphate compounds (pesticide)	N/A	Yes	12
28	PDA/AgNP	Pesticide P_1_	194.76 ppm	Yes	This work
		Pesticide P_2_	114.45 ppm		
		Pesticide P_3_	101.1 ppm		

PDAs are smart conjugate colour-responsive materials that can be easily formed by UV irradiation upon the photopolymerization of diacetylene (DA) supramolecules without any chemical catalyst/inhibitor or any sophisticated system.^13,14^ Owing to the conjugated polymeric ene-yne structure of the PDAs, a strong inter-chain interaction prevents the rotation of the side chain, leading to a significant π-orbital overlapping in the ene–yne backbone. As a result, PDAs absorb the visible radiation and usually turn blue in colour.^15^ In response to various external stimuli such as temperature,^16^ pH,^17^ mechanical stress,^18^ solvents,^19^ metal ions,^20^ and biomolecules,^13^ the blue PDA changes its colour to the purple/red colour that can be observed with the naked eye.^13,15,21^ Because of this unique and intriguing colour changing properties, over the decades, PDAs have been exploited to develop different types of sensors such as biosensors,^13,22^ chemosensors^23^ and other optical sensing applications.^24^ By the incorporation of PDAs with other matrix molecules, or head group structural modification in DA monomers, they can be tuned to interact more strongly with specific target analytes, resulting in colour transformation.^25,26^ This provides selectivity towards target analytes. In this study, green-synthesized AgNPs were incorporated into a PDA matrix for better sensitivity and selectivity. The combination of PDAs with nanomaterials shows great potential for developing eco-conscious sensing platforms. In particular, the mixing of green-synthesized AgNPs with PDAs offers a promising path for pesticide detection. The designed sensor was tested with three locally used pesticides, and satisfactory results were obtained. The proposed sensor is like a litmus paper, and its colour change indicates the presence of pesticides. This is a passive sensor and requires no power supply. Accordingly, it is economic and easy to use without the requirement of any instrument or expert operation. Thus, it can minimize several limitations that exist with the sophisticated pesticide detection techniques. Such sensors not only can address the environmental concern, but also can lay a strong foundation for sustainable sensor technology.

## 2 Experimental section

### 2.1. Materials

10,12-Henicosadiynoic acid (HCDA), analytical grade AgNO_3_ and saponite clay (SC) were purchased from Sigma-Aldrich and used as received. Methanol (spectroscopic grade; (SRL, India) was used as the solvent to prepare the HCDA solution. Distilled water was used to prepare the AgNP solution. Cypermethrin (IUPAC Name: cyano-(3-phenoxyphenyl)methyl] 3-(2,2 dichloroethenyl)-2,2-dimethylcyclopropane-1-carboxylate) (P_1_), pretilachlor (IUPAC Name: 2-chloro-*N*-(2,6-diethylphenyl)-*N*-(2-propoxyethyl)acetamide) (P_2_) and chlorpyriphos/cypermethrin (IUPAC Name: *O*,*O*-diethyl *O*-(3,5,6-trichloro-2-pyridyl) phosphorothioate/cyano-(3-phenoxyphenyl)methyl] 3-(2,2 dichloroethenyl)-2,2-dimethylcyclopropane-1-carboxylate) (P_3_) were purchased from a local market of Agartala, India. The molecular structures of the used materials are shown in Fig. S1 of the ESI.†

### 2.2. Preparation of the HCDA solution

A stock solution of HCDA was prepared by mixing DA monomers into methanol. Following the mixing process, the solution was kept under a cyclomixer for at least 10 minutes. Subsequently, the solution was filtered through a 0.2 μm (PTFE) Teflon filter. The concentration of the active solution was 0.5 mg ml^−1^. As the solution is UV sensitive, the container of the solution was enveloped with aluminum foil. The prepared solution was then stored in a refrigerator for further use.

### 2.3. Synthesis of biogenic AgNPs

The green synthesis process was carried out, as reported in the literature.^47^ To prepare the plant extract, the leaves of *A. heterophylla* were cleaned thrice with distilled water to remove the dust. A total of 10 g of finely chopped leaves were mixed with 100 mL of distilled water and boiled at 70 °C for a duration of 30 minutes. The cooled extract was then filtered using Whatman filter paper (No-1). The resulting light yellow solution was then frozen and stored at 4 °C for further use. The AgNPs were synthesized by reducing a 1 mM AgNO_3_ solution in the presence of *A. heterophylla* leaf extract. Then 90 mL of the 1 mM AgNO_3_ solution was mixed with 10 mL of *A. heterophylla* aqueous leaf extract in a ratio of 9/1 and incubated under ambient conditions under sunlight for 30 minutes. In order to obtain pure AgNPs, the suspension was centrifuged twice at 15 000 rpm for 30 minutes, resulting in a dark brown precipitate that was washed twice with distilled water. After that, the precipitated powder was dried to obtain AgNPs. Several experimental parameters were changed to optimize the conditions for the synthesis of NPs using *A. Heterophylla* leaf extract. Various characterization techniques were used to determine the size and formation of the resulting AgNPs, considering the extract concentration, contact time, and AgNO_3_ concentration.

### 2.4. Characterization of AgNPs

The formation of AgNPs was monitored using a UV-Vis absorption spectrophotometer (Shimadzu, UV 1800) in the range of 200–800 nm. Field Emission Scanning Electron Microscopy (FESEM) (Sigma 300, Zeiss) at 15 kV and Energy Dispersive X-ray (EDX) analysis confirmed the surface morphology and chemical composition of AgNPs. In addition, Dynamic Light Scattering (DLS) analysis was performed using a DLS instrument (Anton Parr, Austria) and a zeta sizer (Nano-ZS, Malvern Instruments, UK) to determine the size, charge distribution and stability of the particles. Moreover, to determine the crystalline nature and phase identification of the synthesized AgNPs, the experiment was conducted using an X-ray diffractometer (SmartLab 9 kW, Rigaku, Japan) with Copper K-alpha (1.54 Å) as an X-ray source. Biomolecules from *A. heterophylla* leaf extract stabilizing AgNPs were identified using Fourier-transform infrared (FTIR) analysis.

### 2.5. Preparation of the clay solution

The experimental procedure involved the use of a SC solution with a concentration of 200 ppm. To prepare this solution, 40 mg of SC was carefully mixed with 200 mL of distilled water. Following that, the resulting mixture was continuously stirred for 24 hours using a magnetic stirrer set at 400 rpm to ensure the formation of a homogeneous and well-dispersed clay solution.

### 2.6. UV-Vis absorption spectroscopy

UV-Vis absorption spectra of pure HCDA and HCDA/AgNP mixed self-standing films were recorded on a hydrophobic quartz glass slide using a UV-Vis absorption spectrophotometer (Shimadzu, UV 1800).

### 2.7. Fabrication of HCDA/clay and HCDA/AgNP/clay-coated paper-based sensors

Pure HCDA and HCDA/AgNP mixed solutions were taken and 200 ppm SC solution was added to those solutions. The solution was held under sonication for 45 minutes to ensure the homogeneous mixing of clay. The solution was then deposited onto a membrane paper (nitrate cellulose, diameter = 47 mm, pore size = 0.22 micron) using a self-standing technique.^19,22,48^ This film was formed by suction-filtration of an aqueous dispersion of HCDA/SC and HCDA/AgNP/SC mixture through the membrane paper. Before exposure to pesticides, a UV light of 254 nm was irradiated for 10 seconds to polymerize the HCDA monomer to get its blue form.

### 2.8. Preparation of the real pesticide sample

The designed sensors were tested with real samples collected from an agricultural field, for which 10 grams of soil sample was taken from the field and mixed with 15 milliliters of acetonitrile, and the mixture was then rapidly shaken for half an hour. Anhydrous MgSO_4_ and NaCl were added to the mixture and centrifuged at 4000 rpm for 10 minutes to remove the moisture. Lastly, to obtain a clean extract for examination, the supernatant was filtered through a 0.22 μm PTFE membrane.^49^

## 3 Results and discussion

### 3.1. Synthesis and characterization of AgNPs

**Fig. 1:**
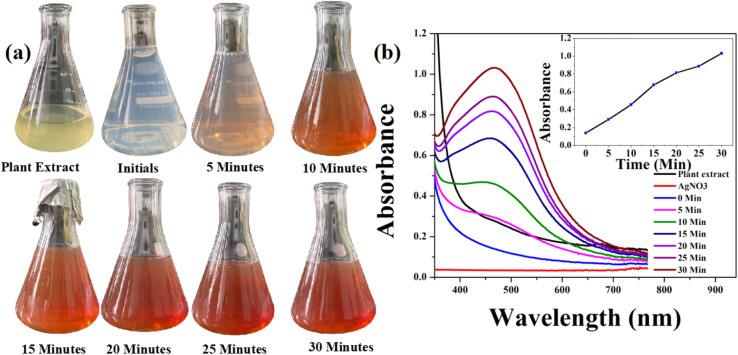
Formation of biosynthesized AgNPs: (a) colour change profile during the formation of AgNPs and (b) time-dependent UV-Vis absorption spectra of the synthesized AgNPs. Inset: plot of the absorbance (466 nm) of AgNPs *versus* incubation time.

The changes in particle size and shape of NPs can be easily analyzed by UV-Vis absorption spectroscopy, providing valuable insights into the formation of complexes.^50^ It is the most common technique to identify the bioreduction of AgNO_3_ into AgNPs. The size, shape and morphology of the NP play an important role in surface plasmon resonance (SPR). Therefore, in the present case, the synthesis and formation of AgNPs using *A. heterophylla* leaf extract were analyzed by UV-Vis absorption spectroscopy.^47,51^ The synthesis process was also demonstrated by a visible colour change from light green to brown over a period of 30 min (Fig. 1a). The UV-Vis absorption studies showed the occurrence of typical SPR throughout the reaction period with an absorption peak at 466 nm ^52^ (Fig. 1b). The intensity of the absorption, as well as the colour, gradually increases with the incubation time (Fig. 1a). The increase in absorbance with incubation time is caused by an increase in the formation of NPs^47,50–52^ [inset of Fig. 1b]. Here the secondary metabolites and other antioxidants present in *A. heterophylla* leaf extract acted as reducing, capping and stabilizing agents in the formation of AgNPs.^47,50,51^ The observed colour change as well as increase in absorbance (∼466 nm) clearly indicates the formation of AgNPs.^47,50–52^

#### FTIR analysis

After successful synthesis of AgNPs using *A. heterophylla* leaf extract, an effort was further made to understand the role of functional groups in the interaction of metal particles with biomolecules, which leads to the reduction of Ag^+^ ions. Moreover, to identify the probable extract component that might help stabilizing and capping the AgNPs, the FTIR spectra of *A. heterophylla* leaf extract and synthesized AgNPs were analyzed [Table 2]. The corresponding FTIR spectra are shown in Fig. S2 of the ESI.† The representative FTIR spectra of the plant extract and synthesized NPs show a number of major peaks positioned at different wavelengths, as summarized in Table 2.

**Table 2:** FTIR analysis of AgNPs synthesized from *A. heterophylla*

Peaks present in the plant extract		Peaks present in the synthesized AgNPs	
Wavenumber (cm^−1^)	Probable functional group	Wavenumber (cm^−1^)	Probable functional group
3261	–OH stretching vibration with polyphenols present in the plant^53^	2914	–CH stretching vibration^53^
2928	–CH stretching vibration^53^	2381	O=C=O stretching vibration of carbonyl bond group^54^
2352	C–O stretching vibrations^55^	2313	O=C=O stretching vibration of carbonyl bond group^54^
1600	N–H bending of amine^56^	1613	Stretching mode of the carboxylate group (COO−)^56^
1388	N–H bending of amide III^57^	1513	C=C aromatics^58^
1238	Vibration of the C–O group of the hydroxyl flavonoids^59^	1371	C–N stretching vibrations^60^
1019	C–N stretching vibrations of primary amines^61^	1220	C–N stretching vibrations^62^
774	Originate from the out-of-plane deformational C–H vibrations indicating the presence of polyphenol^63^	1017	C–N stretching vibrations of primary amines^61^
671	C–C ring stretching vibration^64^	772	Originate from the out-of-plane deformational C–H vibrations indicating the presence of polyphenol^63^
628	C–C ring stretching vibration^64^	618	C–X– stretching vibration of chloride group

The FTIR spectrum of the plant extract showed several distinct absorption bands (Table 2). These absorption bands are related to various functional groups present in the plant extract. A broad absorption peak is observed at 3261 cm^−1^ corresponding to the –OH stretching vibration. This peak is typically associated with the presence of polyphenols, which are known for their antioxidant properties and play a critical role in the reduction of metal ions during nanoparticle synthesis.^53^ Another significant peak is observed at 2928 cm^−1^, corresponding to the –CH stretching vibrations, which indicate the presence of aliphatic hydrocarbons within the plant extract.^53^ The absorption peak at 2352 cm^−1^ is ascribed to the C–O stretching vibrations, suggesting the presence of carbonyl compounds that may participate in the reduction and stabilization of AgNPs.^55^ Additionally, a peak at 1600 cm^−1^ is assigned to N–H bending vibrations, indicating the presence of amine groups, which are potential capping agents for the NPs.^56^ The peak at 1388 cm^−1^ corresponds to the N–H bending vibrations of Amide III, suggesting the presence of proteins or peptides that could contribute to the stabilization of the synthesized NPs.^57^ The FTIR spectrum further shows a peak at 1238 cm^−1^ due to the vibration of the C–O group of hydroxyl flavonoids, known to facilitate the reduction of metal ions during NP synthesis.^59^ The C–N stretching vibrations of primary amines are observed at 1019 cm^−1^. This indicates the presence of nitrogenous compounds that may influence the NP formation.^61^ Additionally, the peaks at 774 cm^−1^ and 671 cm^−1^ are associated with the out-of-plane deformation of C–H vibrations and C–C ring stretching vibrations, respectively. This confirmed the presence of polyphenols and aromatic compounds in the plant extract.^63,64^

The FTIR spectrum of the synthesized AgNPs shows significant shifts and changes compared to the plant extract (Fig. S2 of the ESI† and Table 2). The peak corresponding to the –CH stretching vibrations shifts slightly from 2928 cm^−1^ in the plant extract to 2914 cm^−1^ in the AgNPs, which suggests the involvement of aliphatic hydrocarbons in the NP synthesis.^53^ Notably, the spectrum shows a new peak at 2381 cm^−1^ that corresponds to the O=C=O stretching vibrations of carbonyl bond groups. This suggests that the carbonyl compounds present in the plant extract contribute to the stabilization of AgNPs.^54^ Additionally, the appearance of a peak at 1613 cm^−1^ is associated with the stretching mode of the carboxylate group (COO^−^), which indicates that carboxylate ions are binding to the surface of the AgNPs. The presence of COO^−^ enhances the stability of synthesized NPs.^56^ The spectrum also shows a peak at 1513 cm^−1^ corresponding to C=C aromatic stretching, indicating the role of aromatic compounds in the stabilization of the NPs. Another notable peak appearing at 1371 cm^−1^ corresponds to the C–N stretching vibrations, which suggests that amine groups from the plant extract are involved in the capping of NPs.^61^ Additionally, the C–N stretching vibration observed at 1220 cm^−1^ also indicates the presence of amine groups that are involved in the capping of NPs.^62^ The C–N stretching vibrations of primary amines observed at 1017 cm^−1^ further support the role of nitrogenous compounds in NP formation.^61^ The peak at 772 cm^−1^ is consistent with that for the plant extract, which indicates the presence of polyphenols. These polyphenols play a key role in the reduction and stabilization of AgNPs.^63^ The new peak at 618 cm^−1^ is attributed to the C–X stretching vibration of the chloride group, possibly involved in the NP formation process.

One of the most significant observations is the complete disappearance of the broad absorption band at 3261 cm^−1^ in the FTIR spectrum of the AgNPs, which was present in the plant extract. This disappearance shows that the hydroxyl groups, particularly from polyphenols, are actively consumed during the reduction of Ag^+^ to AgNPs. The absence of this peak in the AgNPs' spectrum indicates that –OH groups likely served as electron donors. These electron donors help in the reduction of Ag^+^ to Ag^0^ and play an important role in the stabilization of the NPs.

The FTIR analysis provides evidence of the successful synthesis of AgNPs using the plant extract. The observed shifts, appearance and disappearance of specific absorption bands in the FTIR spectrum of the synthesized AgNPs compared to the plant extract confirm the active participation of various functional groups in the reduction and stabilization processes. The complete disappearance of the 3261 cm^−1^ peak indicates the critical role of hydroxyl groups. These hydroxyl groups are particularly from polyphenols that served as both reducing agents and stabilizers for the AgNPs.

#### FESEM and EDX analysis

**Fig. 2:**
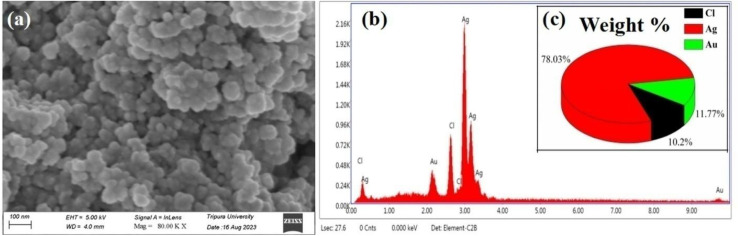
(a) FESEM images of the biosynthesized AgNPs using *A. heterophylla* leaf extract. (b) EDAX spectrum of the synthesized AgNPs. (c) Weight percentage of the elemental composition of the biosynthesized AgNPs obtained from EDX analysis.

FESEM has been employed to have a visual idea about the shape and morphology of the synthesized AgNPs. The FESEM images show that AgNPs were successfully synthesized by a green synthesis method with *A. heterophylla* leaf extract acting as a natural reducing and stabilizing agent (Fig. 2a). The NPs are spherical and well dispersed, with an average particle size ranging from 60 to 80 nm. This result is consistent with those reported in the literature.^65^ Furthermore, EDX analysis was performed to have an idea about the elemental composition of the synthesized AgNPs^66^ [Fig. 2b]. The EDX results revealed the presence of a prominent peak at 3 keV, which indicates the presence of silver in the AgNPs. The intensity and weight percentage of the peak at 3 keV indicate Ag as the main component. This result is in good agreement with the reported results.^66^ The Au peak is due to the conducting metallic surface coating during sample preparation for FESEM measurement. The low-intense peak of Cl may be due to the capping effect of biomolecules that were present in *A. heterophylla* leaf extract during AgNP synthesis. Cl also might have been diffused within the AgNPs because of their relatively high diffusion coefficients and very small ionic radius.^67^ Such elements often appear in a variety of metallic and oxide biosynthesized substances.^66^Fig. 2c demonstrates the quantitative analysis that reveals the weight percentage of each individual constituent in the sample.

#### TEM analysis

**Fig. 3:**
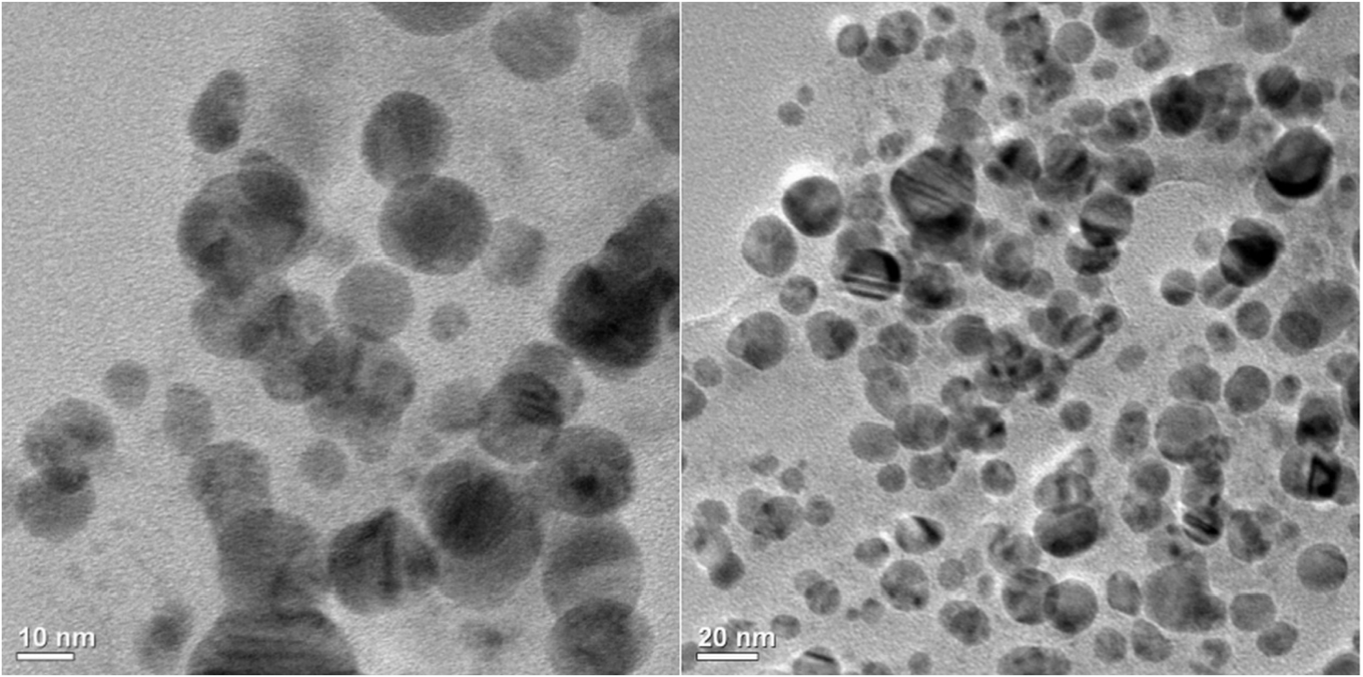
TEM images of the synthesized AgNPs using *A. heterophylla* leaf extract.

To investigate the morphology and size distribution of the synthesized AgNPs, TEM analysis was performed, as shown in Fig. 3. The images revealed that the AgNPs are found to be quasi-spherical in shape. The particle size distribution analysis indicated an average diameter of approximately 16.16 nm, with sizes ranging between 4.025 and 29.95 nm. The observed size range confirms the nanoscale nature of the synthesized AgNPs. The spherical shape of the synthesized AgNPs as observed in the TEM images is similar to that revealed by FESEM analysis.

#### DLS and zeta potential analysis

**Fig. 4:**
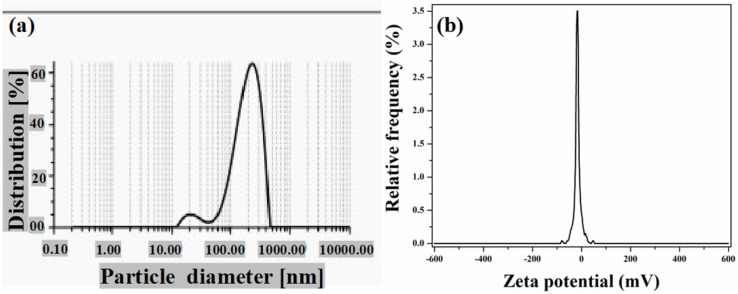
(a) DLS spectra of the biosynthesized AgNPs. (b) Zeta potential spectra of the biosynthesized AgNPs.

DLS analysis was also performed to find out the hydrodynamic size and polydispersity index (PDI) of the biosynthesized AgNPs in a colloidal aqueous form. Fig. 4a demonstrates the size distribution of green-synthesized AgNPs ranging from 22 to 208 nm. The average hydrodynamic diameter was 169.04 nm. The PDI value of AgNP was 0.265. The PDI value ‘0’ indicates monodisperse distribution, while the value ‘1’ indicates polydisperse distribution.^68^ Moreover, in order to get the idea about the surface charges and stability, zeta potential analysis was conducted. The zeta potential value of the synthesized AgNPs was found to be −16.93 mV [Fig. 4b]. The negative value of zeta potential indicates that the AgNPs are moderately stable and prevent the aggregation due to electrostatic repulsion. The negative surface charge is probably due to the adsorption of negatively charged functional groups obtained from *A. heterophylla* leaf extract.

#### XRD analysis

**Fig. 5:**
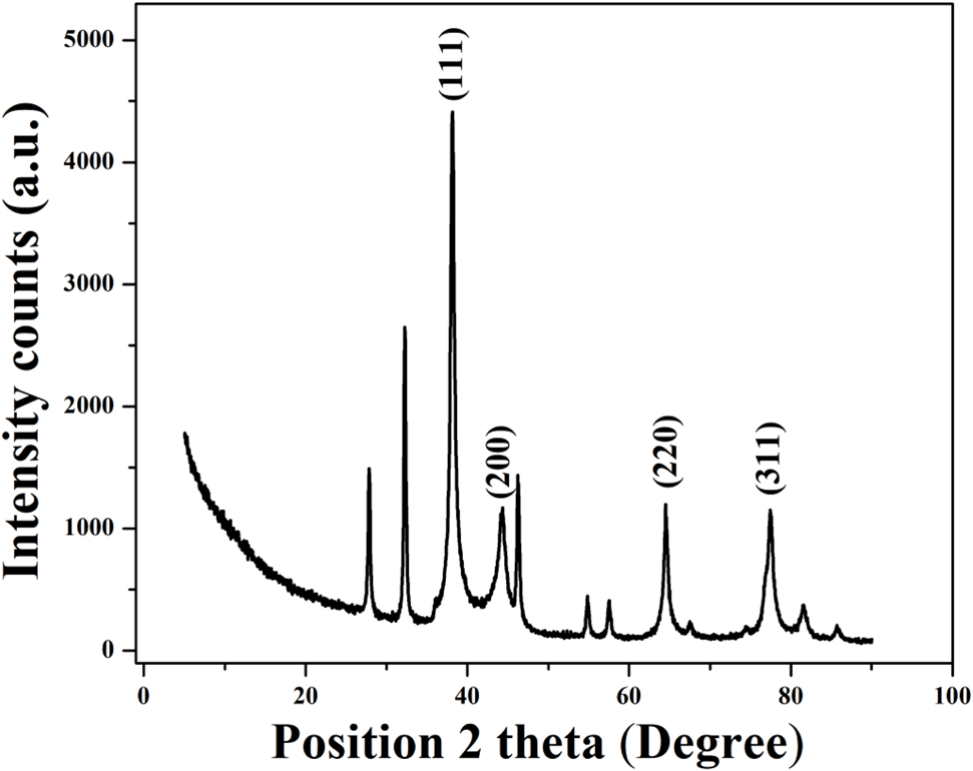
X-ray diffraction pattern of the synthesized AgNPs.

XRD analysis was performed to determine the crystalline nature and phase identification of the synthesized AgNPs. The diffraction pattern (Fig. 5) shows distinct peaks at 2*θ* values of 38.146°, 44.42°, 64.51°, and 77.46°, corresponding to the (111), (200), (220), and (311) planes of face-centered cubic (FCC) silver (JCPDS no. 04-0783).^69^ The intense peak at 38° (111) suggests a strong preferential orientation along this plane, which is a characteristic feature of nanoscale silver.

The crystallite size was calculated using Debye–Scherrer's equation: *D* = 0.9*λ*/*β* cos *θ*, where *D* is the crystallite size, *λ* indicates the wavelength of Cu-Kα X-ray radiation (1.5406 Å), *β* is the full width at half maximum (FWHM) in radians, and *θ* is the Bragg angle. The average calculated crystallite size was found to be 47.70 nm [Table 3]. The presence of other additional minor peaks may indicate the presence of residual biomolecules from the plant extract. Overall, the XRD results confirmed the successful synthesis of highly crystalline AgNPs with an FCC structure.

**Table 3:** Calculation of the crystal size of the AgNPs from the X-ray diffraction pattern using the Scherrer equation

Peak Pos. [°2*θ*]	FWHM left [°2*θ*]	*d*-spacing [Å]	Miller indices (*hkl*) plane	Crystallite size (nm)	Average crystallite size (nm)
38.146	0.1023	2.35922	111	82.17	47.70
44.420	0.5628	2.03949	200	15.25	
64.510	0.1248	1.44336	220	75.27	
77.468	0.5616	1.23108	311	18.13	

Therefore, it can be concluded that typical AgNPs were formed. However, differences in particle size were observed among the results from FESEM, TEM, DLS, and XRD. It may be noted that electron microscopy and DLS are two different techniques and work on different principles. The DLS sizes depend on the particle physical model, whereas the FESEM sizes depend on the interaction between electron beams with the sample. Therefore, it is difficult to compare the particle sizes measured by FESEM and DLS. DLS is an indirect method that depends on the optical environment, which can lead to differences in particle size. DLS measures the hydrodynamic diameter of a particle that includes the ionic shell that surrounds the particle. This can significantly affect the measured particle size.^46^ In the present case, DLS measurement was done for AgNPs in water dispersion. Therefore, water molecules may surround the NP leading to an increase in hydrodynamic diameter. Despite these discussed variations in particle size arising from the different analytical techniques, the obtained results are in close agreement with previously reported findings.^70^

The differences in particle sizes across these techniques are due to the unique principles of each technique. While TEM and FESEM measure the dry core particle size, DLS provides the hydrodynamic diameter, which includes surface-bound phytochemicals and solvation layers. XRD, however, estimates the crystallite size. Such differences in the size of NPs are well reported.^71^

In the preceding section of the manuscript, we have discussed the synthesis as well as characterization of AgNPs. However, the main objective of this study is to investigate the effect of such NPs on the colorimetric phase behaviour of PDA, so that the PDA-AgNP system can be applied towards designing pesticide sensors. The results of the corresponding investigation have been reported in the following section.

### 3.2. Role of saponite clay (SC)

In this work, we designed a colorimetric paper-based sensor using a PDA/AgNP system following a self-standing technique.^19,22,48^ A nitrate cellulose membrane of pore size 0.22 micron was used. It has been observed that the fabrication of self-standing films with pure HCDA or a mixture of HCDA and AgNPs results in limited incorporation of molecules into the membrane. Hardly, a uniform film was deposited. Moreover, the stability of the film was very poor. Therefore, SC has been used to improve the incorporation of HCDA molecules and AgNPs into the membrane paper. In the presence of SC, a uniform film was deposited onto the membrane and the film could be easily transferred onto a glass substrate.^19,22,48^ The intercalation as well as the cation exchange capabilities of SC allows it to be incorporated between clay layers *via* intercalation and cation-exchange reactions. Moreover, it has been observed that by the incorporation of clay in the sensor, the mechanical stability of the sensor increases even beyond 200 days.

### 3.3. Phase behaviour of PDAs

The unique behaviour of PDA with two distinct chromatic (blue and red) phases makes them potential sensing elements to design low-cost colorimetric sensors.^13,14,20,22,23^ In such sensors, the sensing is done through marked colour transition from blue to red. Such colour transition can be seen through naked eyes.^13,16,18–20,22,72^ Therefore, tuning the colour-transition behaviours of PDA-based materials is essential to demonstrate their full potential towards designing various sensors. One of the best methods is the structural modification of PDAs or mixing PDAs with suitable matrix materials that helps the PDAs to manipulate the colour transition in response to the target analytes.^25,26^ Combining PDAs with other matrix materials sometimes improves or changes PDA properties towards the detection of specific target analytes.^21,25,26^ This results in the development of a novel sensing system with improved sensitivity and selectivity. DA derivatives show multiple distinct phases during polymerization.^25,26^ Therefore, it is important to understand and optimize the PDA phases. It has been observed that the sensitivity and selectivity towards a target analyte highly depends on the matrix material as well as its mixing ratio with PDA. Therefore, the optimization of the appropriate mixing ratio of PDAs with matrix materials is highly important.

In the present case, it has been tried to optimize the phase behaviour of PDAs mixed with AgNPs to design a pesticide sensor. In order to study the phase behavior of the pure PDA and PDA/AgNP mixed system, the self-standing films were transferred onto hydrophobic quartz glass substrates and exposed to external stimuli including UV light and heat for varying duration. Corresponding UV-Vis absorption spectra and colour change of the film have been recorded. The UV-Vis absorption spectra of the pure HCDA, AgNP and HCDA/AgNP mixed self-standing films for three different mixing ratios were investigated upon exposure to UV light as well as temperature. The UV-Vis absorption spectra of pure AgNPs under various UV-exposure doses are shown in Fig. S3 of the ESI.† Pure AgNPs show the absorption band at 472 nm in the absence of UV exposure, which is slightly shifted with respect to the same in liquid dispersion [Fig. 1b, S3 of the ESI†]. This slight shift in peak position may be related to differences in the aggregation states of NPs when transferred onto a solid substrate.^73^ Upon UV exposure, the absorption spectrum of AgNPs hardly shows any change [Fig. S3 of the ESI†]. The absorption spectra of the AgNP film remained almost the same. This shows that the optical characteristics and overall appearances of the NPs remained almost unaffected upon UV exposure. This also indicates very good stability or lack of any significant UV-induced alterations of AgNPs.

**Fig. 6:**
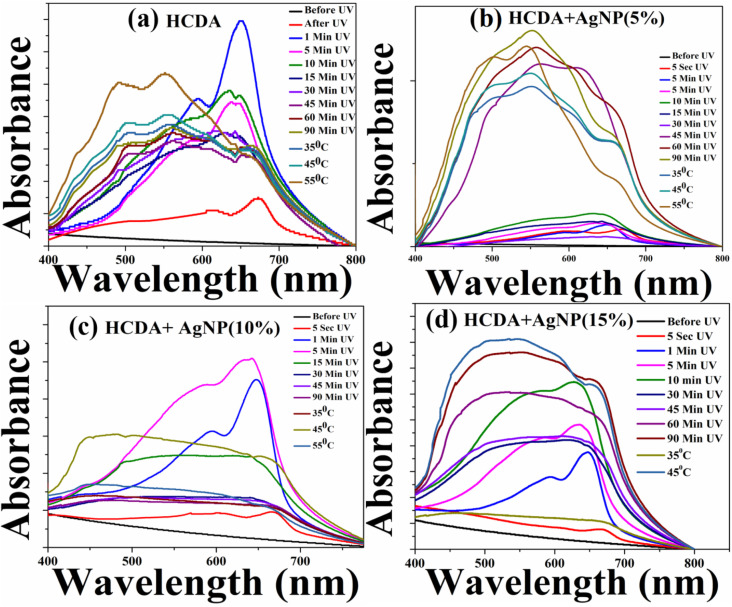
UV-Vis absorption spectra of (a) pure HCDA, (b) HCDA/AgNP (5%), (c) HCDA/AgNP (10%), and (d) HCDA/AgNP (15%) upon UV-irradiation and heat treatment. The exposure time is given in the inset.

The UV-Vis absorption spectra of the pure HCDA and HCDA/AgNP mixed film of 5%, 10%, and 15% of AgNPs upon UV-irradiation, followed by the heat treatment, are shown in Fig. 6a–d, respectively. It has been observed that prior to UV exposure, *i.e.* before polymerization, HCDA did not absorb any light due to the lack of conjugated double bonds that are required for excited-state electronic transitions.^74^ After 5 seconds of UV irradiation, an absorption band has appeared at 673 nm [Fig. 6a]. The absorption band is associated with the blue phase of PDA.^13,22^ This indicates that the HCDA monomer has begun to polymerize. PDA blue peak originates due to the rotation polymeric backbone accompanied by the straightening of an alkyl chain of HCDA that effects the overall conformation of the polymer backbone from planar to nonplanar configuration.^15^ The absorption peak shifted to 650 nm with a hump at 594 nm upon 1 minute of UV irradiation. These absorption peaks are also associated with the blue phase of PDA.^19,22,75^ It is obvious from Fig. 6a that with 1 minute of UV irradiation, the absorbance has become maximum. The blue phase peaks started to shift towards the red phase when the UV exposure time was increased further. After 45 minutes of UV exposure, the absorption band shifted to 562 and 502 nm, leaving a trace of blue PDA with 652 nm absorption peak [Fig. 6a]. These 562 nm and 502 nm bands correspond to PDA's red phase.^13,22,23,26,64^ This indicates that at this stage both blue and red phases of PDAs co-exist. The reason behind the shifting of absorption band from 674 nm to 562 nm is that when UV light is irradiated on PDA, the high-energy photons got absorbed by the PDA. The absorbed UV energy stimulates electrons along PDA-conjugated-bonds, allowing them to transit from the ground state to higher energy states. The excited electrons of PDA take part in the photochemical reaction known as photo-polymerization. Prolonged exposure to UV radiation causes thermochromism.^76,77^ Moreover, the photo-polymerization results in the formation of longer polymer chains, altering the molecular structure of PDA. The photopolymerization reaction increases the conjugation length of PDA chains as well. Longer conjugation lengths cause the shift of the absorption spectra. When the UV exposure increased further, the intensity corresponding to blue phase of PDA gradually decreased, while the intensity of absorption peak corresponding to red phase was increased. After 90 minutes of exposure, there was no further change in spectral profile upon increase in UV exposure time. Both blue and red phases co-existed at this stage. Therefore, complete transition from blue to red form of PDA was not possible upon UV exposure. However, upon temperature treatment, it has been observed that complete transition from blue phases of PDA to its red phase occurred.

The initial phase behaviour of HCDA in the presence of AgNPs was almost similar to that of pure HCDA. Here, in the case of three mixing ratios, it was observed that before UV exposure HCDA remained as a monomer with almost no absorption.^22^ The corresponding absorption spectra are shown in Fig. 6b–d, respectively, for 5%, 10% and 15% of AgNPs in the HCDA/AgNP system. Upon UV exposure initially, the absorption peaks due to the blue phase of PDA appeared, which increases with UV exposure time. Although, after certain exposure time, peaks due to the red phase slowly appeared at the expense of blue phase absorption peaks. However, for complete transition from blue PDA to red PDA, temperature treatment was required. The interesting thing is that here the onset of blue as well as red phase of PDA was strongly dependent on the percentage of AgNPs in the HCDA/AgNP system. The phase behaviour of PDA in the presence of 10% of AgNPs was very interesting (Fig. 6c and Table 4). Here, the intensity of the peak corresponding to the blue phase increases significantly with a shift of peak from 667 nm to 647 nm after 1 min of UV exposure. The increase in absorbance shows the extent of polymerization of HCDA molecule in the film to its blue phase. The absorbance of the peak at 643 nm becomes maximum after 5 min of UV exposure. This indicates that most of the HCDA molecules in the HCDA/AgNP (10%) mixed film had polymerized to its blue form [Fig. 6c]. When the UV dosage time is gradually increased interestingly after 15 minutes of UV exposure, the peak at 574 nm corresponding to the red phase appeared, whereas pure HCDA required 45 min to start blue-to-red transition. The onset of blue-to-red PDA phase transition at earlier time indicates that the PDA is more sensitive to the analytes in this system. This clearly shows the effect of AgNPs towards the phase change behaviour of PDAs. Such behaviour is probably due to the photodegradation process caused by the strongly enhanced photoexcitation.^41^ It has been reported that DA chains are densely arranged on the NP surface and form bilayered organization, which might also enhance the potential of the phase changing behaviour.^46^ It may also be mentioned in this context that there may be interaction between the –COOH group of PDA and the –OH group on the AgNP surface in the PDA/AgNP system.^26,78^ This may give rise to the formation of nanohybrids with a core–shell structure, where the ordered layer of PDA molecules may facilitate better exposure of PDA molecules to external stimuli, resulting in improved polymerization.^26^ On further increasing the dosages of UV exposure time, the intensity of blue peak decreases, while the intensity of the red peaks increases as of pure PDA. Information about PDA phase change as extracted from the UV-Vis absorption spectra in the presence of 5%, 10%, and 15% AgNP mixed system and that of pure HCDA are presented in Table 4.

**Table 4:** Information about the phase transition behaviour of HCDA in the presence of AgNPs extracted from the absorption spectra shown in Fig. 6

Sensing system	UV exposure time to convert HCDA monomer to HCDA blue phase	UV exposure time to starting convert blue HCDA to red HCDA	Temperature at which complete blue to red conversion occurred
Pure HCDA	1 min (650 nm and 594 nm)	45 min (561 nm and 502 nm)	55 °C (552 nm and 492 nm)
HCDA/AgNP (5%)	10 min (631 nm and 574 nm)	45 min (615 nm and 558 nm)	55 °C (544 nm and 502 nm)
HCDA/AgNP (10%)	5 min (643 nm and 585 nm)	15 min (628 nm and 550 nm)	55 °C (552 nm and 500 nm)
HCDA/AgNP (15%)	10 min (629 nm and 578 nm)	30 min (625 nm and 551 nm)	45 °C (552 nm and 492 nm)

From Table 4, it is observed that the PDA phase change was faster in the presence of AgNPs than that of pure PDA. Maximum response was observed in the case of HCDA/AgNP mixed system with 10% of AgNP.

### 3.4. Chromatic response (CR) of PDA towards pesticides

It has been observed that the presence of AgNPs has effects on the phase behaviour of PDA. Moreover, the AgNP concentration plays an important role towards PDA phase transition. Therefore, in an effort to design pesticide sensors, pure self-standing films of PDA as well as PDA/AgNP mixed system were exposed to different pesticides, namely, P_1_, P_2_ and P_3_ at different concentrations. Before exposure to pesticides, the self-standing films were transferred onto a quartz glass substrate. DA monomers were polymerized to their blue form upon UV exposure.^14,16,20,23,72^ After that, polymerized blue films have been exposed to pesticides. The UV-Vis absorption spectra were recorded before and after exposure to pesticides. In order to quantify the blue-to-red colour transition of PDA, the UV-Vis absorption spectra were analyzed and % CR was calculated by defining % CR = [(PB_bef_ – PB_aft_)/PB_bef_] × 100%, where PB_bef_ and PB_aft_ are the relative ratios of blue and red elements before and after exposure to pesticides, respectively.^79^ PB can be defined as *A*_blue_/(*A*_blue_ + *A*_red_), where *A*_blue_ and *A*_red_ are the absorbances of the peaks corresponding to blue and red phases, respectively.^79^

**Fig. 7:**
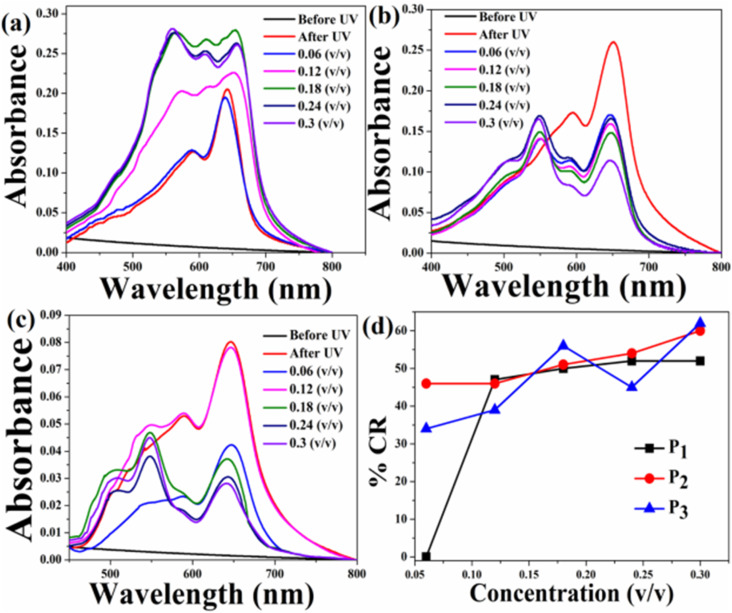
Absorption spectra of the pure HCDA sensing system upon exposure to (a) pesticide P_1_, (b) pesticide P_2_, and (c) pesticide P_3_ at varied concentrations, and (d) % CR of pesticides P_1_, P_2_ and P_3_, as calculated from (a)–(c).

The UV-Vis absorption spectra of the pure PDA-based system before and after exposure to the three pesticides at different concentrations are shown in Fig. 7a–c. Initially, PDAs were converted into their blue forms upon UV exposure. Therefore, before exposure, prominent peaks corresponding to the blue phase appeared and no absorption peaks corresponding to red phase were observed [Fig. 7a–c]. The absorption spectra measured after exposure to three pesticides indicate that the PDA phase changes from blue to red polymers occurred in the presence of pesticides. This was confirmed by the decrease in absorption peaks due to the PDA blue phase and the corresponding increase in the PDA red phase. Here, the PDA responded to all the three pesticides. However, the extent of phase change was strongly dependent on the pesticide concentration. In order to quantify the PDA blue-to-red phase transition, the % CR was calculated for all the three pesticides, as shown in Fig. 7d. From the CR analysis, it was observed that in the case of P_1_, the PDA phase conversion started at a concentration of 0.12 (v/v) and maximum blue-to-red transition was 52% for the concentration of 0.3 (v/v). PDA phase conversion started at 0.06 (v/v) for both P_2_ and P_3_. Maximum blue-to-red conversion was 60% and 62% at a concentration of 0.3 (v/v) for the pesticides P_2_ and P_3_, respectively.

We also exposed the HCDA/AgNP system at different concentrations of AgNPs to three pesticides under investigation. It is expected that here the blue-to-red phase change of PDA might be different compared to the pure PDA. This is because it has been observed that the presence of AgNPs affected the PDA phase behaviour. Before doing that we exposed the pure AgNP film to the pesticides at different concentrations and the corresponding absorption spectra were recorded. However, hardly any change in the spectral profile was observed for all the three pesticides at different concentrations (Fig. S4 of the ESI†). This indicates that hardly any change occurred in the case of pure AgNPs in the presence of pesticides.

**Fig. 8:**
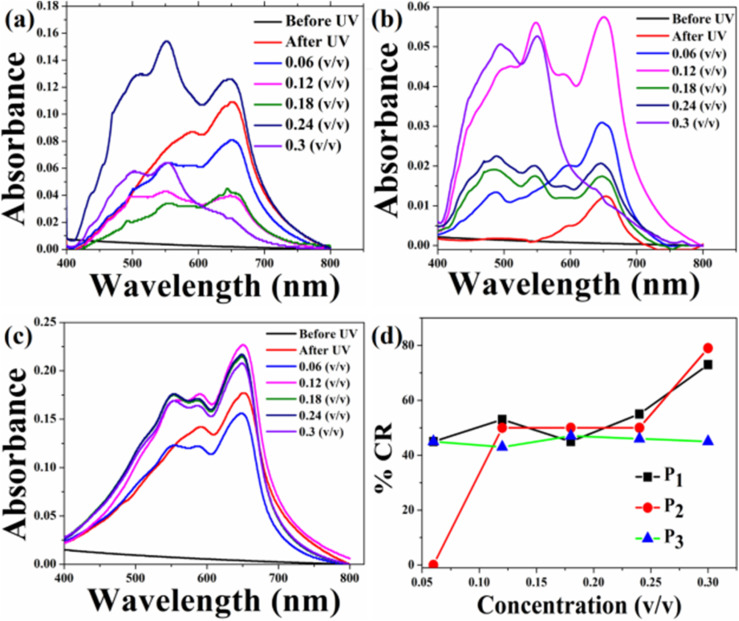
Absorption spectra of the HCDA/AgNP (5%) sensing system upon exposure to (a) pesticide P_1_, (b) pesticide P_2_, and (c) pesticide P_3_ with different concentrations, and (d) % CR of pesticides P_1_, P_2_ and P_3_ as calculated from (a–c).

**Fig. 9:**
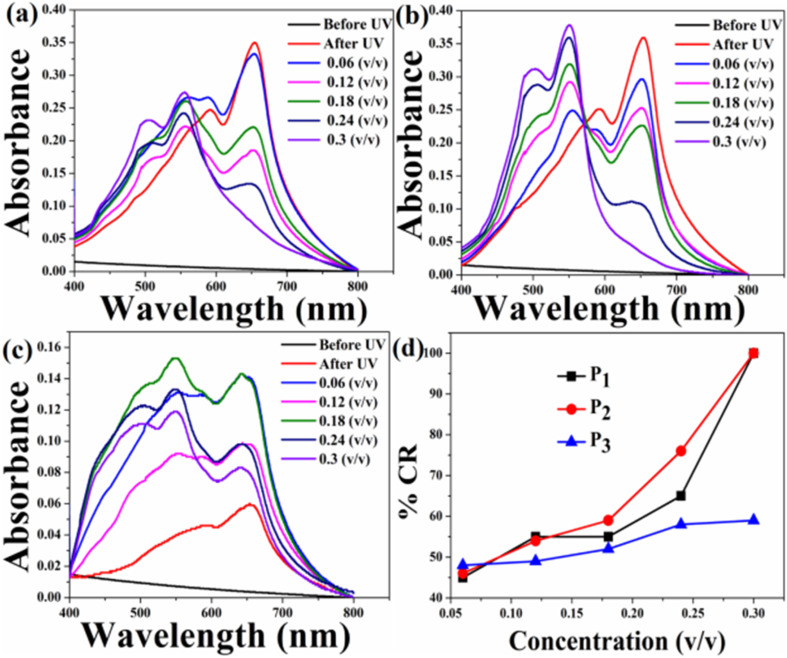
CR and respective absorption spectra of the HCDA/AgNP (10%) sensing system upon exposure to (a) pesticide P_1_, (b) pesticide P_2_ and (c) pesticide P_3_ at different concentrations, and (d) % CR of pesticides P_1_, P_2_ and P_3_ as calculated from (a)–(c).

**Fig. 10:**
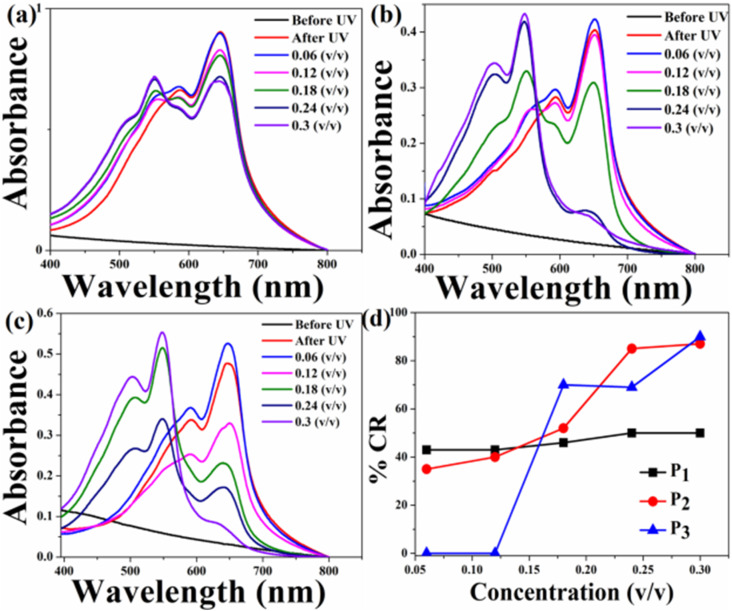
Absorption spectra of the HCDA/AgNP (15%) sensing system upon exposure to (a) pesticide P_1_, (b) pesticide P_2_ and (c) pesticide P_3_ at different concentrations, and (d) %CR of pesticides P_1_, P_2_ and P_3_ as calculated from (a)–(c).

The UV-Vis absorption spectra of the HCDA/AgNP system with 5%, 10% and 15% of AgNPs before and after exposure to three pesticides P_1_, P_2_ and P_3_ at different concentration are shown in Fig. 8, Fig. 9 and 10, respectively. The maximum % CR and PDA phase change starting concentration for all the three HCDA/AgNP systems under investigation are listed in Table 5. It was observed that for all the three HCDA/AgNP systems, blue-to-red phase conversion occurred, although the phase change was strongly dependent on the AgNP concentration in the system and on the concentration and type of pesticides.

**Table 5:** Phase change starting concentrations and maximum % CR for the HCDA and HCDA/AgNP-based sensing system for different pesticides

	Phase change starting concentration (v/v)			Maximum % CR at 0.3 (v/v)		
	P_1_	P_2_	P_3_	P_1_	P_2_	P_3_
Pure HCDA	0.12	0.06	0.06	52	60	62
HCDA/AgNP (5%)	0.06	0.12	0.06	73	79	45
HCDA/AgNP (10%)	0.06	0.06	0.06	100	100	59
HCDA/AgNP (15%)	0.06	0.06	0.18	50	87	90

For HCDA/AgNP (5%), the PDA phase change started for P_1_ and P_3_ at a concentration of 0.06 (v/v) and for P_2_ at a concentration of 0.12 (v/v) [Fig. 8 and Table 5]. The maximum blue-to-red PDA phase change conversion was 73%, 79% and 45% for the three pesticides P_1_, P_2_ and P_3_, respectively.

In the case of HCDA/AgNP (10%), the PDA phase change started at a concentration of 0.06 (v/v) for all the three pesticides and 100% blue-to-red phase conversion of PDA occurred for both P_1_ and P_2_. In the case of P_3_, the same was 59% [Fig. 9 and Table 5].

In the case of the HCDA/AgNP (15%) system, PDA blue-to-red phase conversion started at a pesticide concentration of 0.06 (v/v) for P_1_ and P_2_. In the presence of P_3_, the blue-to-red conversion started at a concentration of 0.18 (v/v). The PDA blue-to-red phase conversion was 50%, 87% and 90%, respectively, in the presence of three pesticides P_1_, P_2_ and P_3_ [Fig. 10 and Table 5].

From the % CR analysis, it has been observed that the pure HCDA-based sensing system showed limited chromatic response (% CR < 64%) and partial blue-to-red phase transition upon pesticide exposure. However, the addition of AgNPs improved the chromatic response, and almost 100% PDA blue-to-red phase change occurred in the presence of pesticides P_1_ and P_2_ when the AgNP concentration was 10% in the HCDA/AgNP system. In the case of pesticide P_3_, the maximum blue-to-red conversion (90%) occurred when the AgNP concentration was 15%.

### 3.5. Design of PDA-based paper sensors

PDA-based paper sensors were prepared by depositing the HCDA or HCDA/AgNP mixed system onto a cellulose membrane following a suction technique.^22,72^ After depositing on the membrane, the HCDA molecules were transformed to their blue form *via* photochemical (UV-exposure) polymerization.^13,14,26,74^ The colour of the paper becomes blue and ready for use. Such sensors are very similar to litmus paper.

Here, we prepared four sets of sensors employing HCDA with different amounts of AgNPs, *viz.* (i) Pure HCDA, (ii) HCDA/AgNP (5%), (iii) HCDA/AgNP (10%) and (iv) HCDA/AgNP (15%). All the four sets of sensors were exposed to three pesticides at different concentrations. The physical demonstration of the sensor is shown in Fig. S5 of the ESI.† From the % CR analysis, it was observed that the HCDA phase change from blue to red occurred in the presence of these pesticides. Based on these phase change, colours of the sensors change from blue to red upon pesticide exposure, although the extent of colour change was different for different sensors and concentrations of pesticides. Such colour change was visible for the naked eyes. However, sometimes, it has become a challenge to understand and quantify the colour change. Therefore, in order to gain better insights into the colorimetric response, RGB analysis of the images of sensors before and after exposure were analyzed using the MATLAB software.^72^ Based on the analysis, the normalized signal intensity (NSI) was calculated using the relation NSI = (*R*/*B*)/(*R*_0_/*B*_0_), where *R*_0_, *B*_0_ and *R*, *B* are the values of the red and blue components before and after exposure to pesticides.^80^

**Fig. 11:**
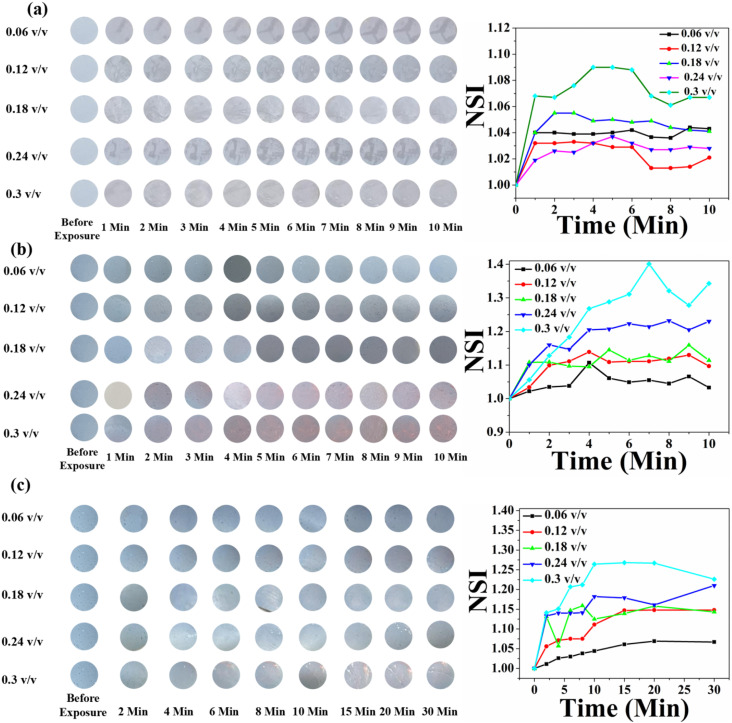
Time- and concentration-dependent colorimetric response of pure HCDA-based paper sensors upon exposure to (a) pesticide P_1_, (b) pesticide P_2_ and (c) pesticide P_3_. The respective NSI values are presented alongside each set of colorimetric transitions, demonstrating the sensor's response across different concentrations and time intervals.

Fig. 11 shows the time- and concentration-dependent colorimetric response of pure HCDA-based paper sensors exposed to (a) pesticide P_1_, (b) pesticide P_2_, and (c) pesticide P_3_. Visible colour changes and corresponding NSI plots show that the sensors responded to the presence of pesticides. However, the extent of response depends on the types of pesticides as well as their concentration and exposure time. From Fig. 11, it was been observed that when the HCDA paper-based sensor was exposed to pesticide P_1_ and P_3_, almost no visual colorimetric change was detected initially, even at higher pesticide concentrations [Panels a and c, Fig. 11]. The lack of response was observed within the first 10 minutes of exposure for all tested pesticides, including concentrations up to 0.3 v/v, though there is a minute increase in NSI values. However, when the sensor was exposed to P_2_ after 1 min, it started to show colorimetric transformation at concentrations of 0.24 (v/v) and 0.3 (v/v) [panel b, Fig. 11]. With the increase in exposure time for P_2_, it was observed that the NSI value also increases. Here also colour change was not observed up to a concentration of 1.8%, while a negligible increase in NSI was observed.

**Fig. 12:**
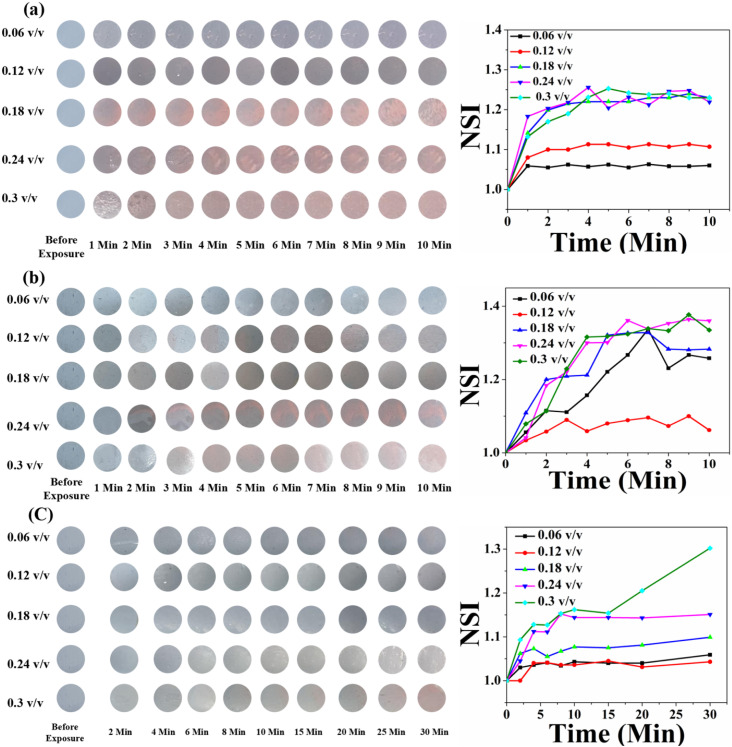
Time- and concentration-dependent colorimetric response to the HCDA/AgNP (5%)-based paper sensor upon exposure to (a) pesticide P_1,_ (b) pesticide P_2_ and (c) pesticide P_3_. The respective NSI values are presented alongside each set of colorimetric transitions, demonstrating the sensor's response across different concentrations and time intervals.

The colorimetric response of the HCDA/AgNP (5%) sensor is shown in Fig. 12. Unlike that of pure HCDA, here marked colour changes were observed for all the pesticides. The colour change becomes prominent at a higher concentration of pesticides with a higher exposure time. In the case of P_1_, the sensor responded at a pesticide concentration of 0.18 (v/v) and higher with an exposure time of 1 min. From the NSI plot (Fig. 12a), it was observed that the sensor responded well to a concentration of 0.18 (v/v) or higher and maximum blue-to-red conversion occurred within 4 min. However, for pesticide P_2_, the sensor showed a visible response with an exposure time of 2–3 min for a concentration of 0.18 (v/v) or higher. Approximately after 6 min of exposure, maximum colour transformation occurred. In the case of pesticide P_3_, the response is less than that of pesticide P_1_ and pesticide P_2_. Here, the sensor responded to a higher concentration with a longer exposure duration.

**Fig. 13:**
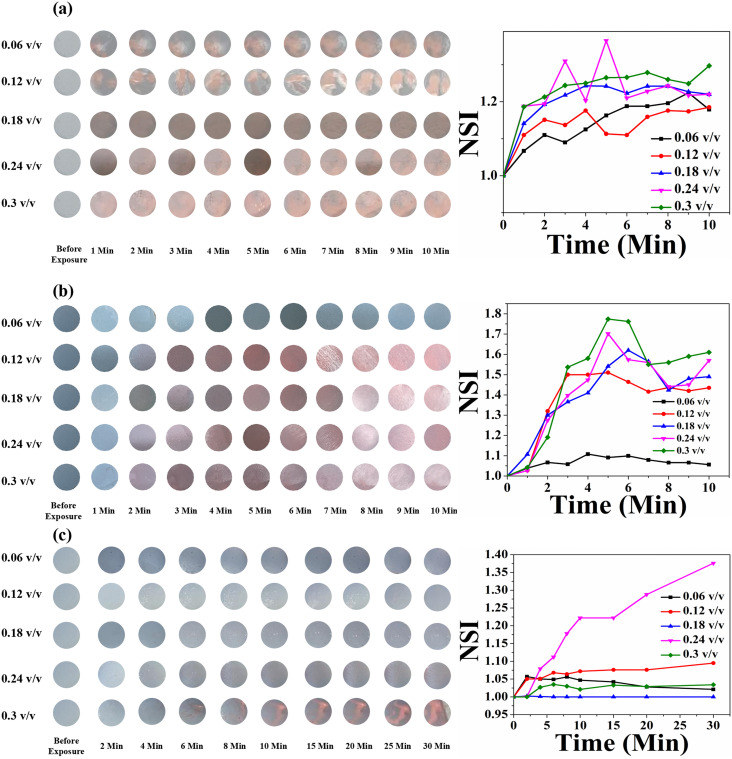
Time- and concentration-dependent colorimetric response of the HCDA/AgNP (10%)-based paper sensor upon exposure to (a) pesticide P_1_, (b) pesticide P_2_ and (c) pesticide P_3_. The respective NSI values are presented alongside each set of colorimetric transitions, demonstrating the sensor's response across different concentrations and time intervals.

In the case of HCDA/AgNP (10%), the colorimetric response was better. Here, the colour change occurred with less exposure time and at a lower concentration. Immediately after exposure (<1 s) and even for 0.06 (v/v) of pesticide P_1_, marked visible colour changes were observed [panel a, Fig. 13]. In the case of pesticide P_2_, the sensor responded to a concentration of 0.12 (v/v) or higher with an exposure time of 2 min [panel b, Fig. 13].

**Fig. 14:**
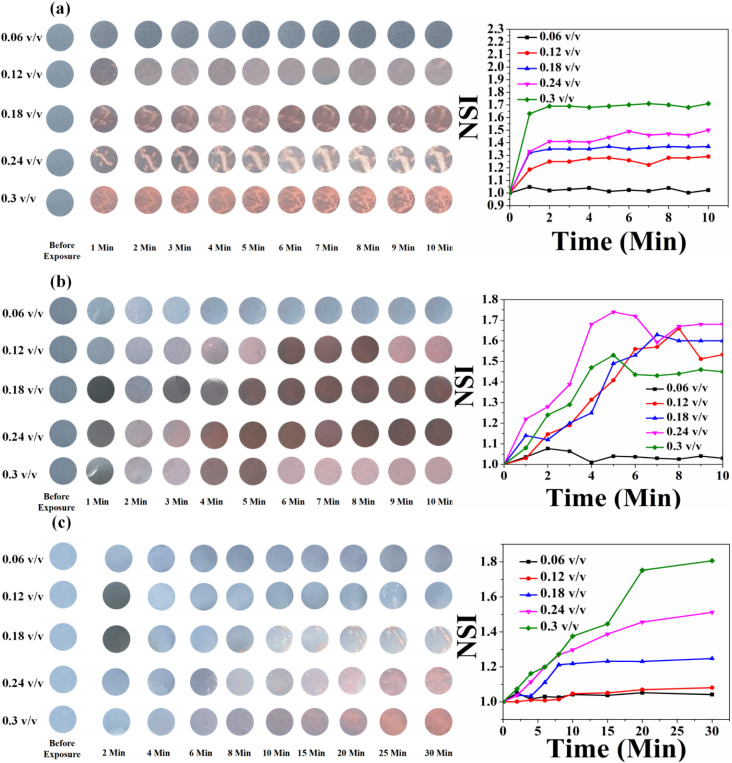
Time- and concentration-dependent colorimetric response of the HCDA/AgNP (15%)-based paper sensor upon exposure to (a) pesticide P_1_, (b) pesticide P_2_ and (c) pesticide P_3_. The respective NSI values are presented alongside each set of colorimetric transitions, demonstrating the sensor's response across different concentrations and time intervals.

However, for pesticide P_3_, the sensor responded well to only a high concentration of 0.3 (v/v) with an exposure time of 5 min [panel c, Fig. 13]. The HCDA/AgNP (10%) sensor showed better response to pesticide P_1_ and P_2_ than all the other sensors under investigation. The limits of detection for P_1_ and P_2_ were calculated as 194.76 ppm and 114.45 ppm, respectively. The limit of detection was calculated based on an earlier report.^81^ The colorimetric responses along with the NSI plot for the HCDA/AgNP (15%) sensor to the three pesticides under investigation are shown in Fig. 14.

It was observed that for pesticide P_1_, the colour change started from the concentration of 0.12 v/v [panel a, Fig. 14]. Additionally, the intensity of the colour change increased with the increase in concentration but reached saturation after 2 min. The plot of NSI *versus* time supported this observation. The extent of colour change was higher than that of the pure HCDA-based sensor, as confirmed by the % CR analysis. For pesticide P_2_, marked colour change started at a concentration of 0.12 v/v [panel b, Fig. 14]. It has been observed that the intensity of the colour change increased with the increase in pesticide concentrations and time but reached saturation after 5–8 minutes. For the detection of pesticide P_3_, the HCDA/AgNP (15%) sensor showed a better chromatic response than that of all the other sensors. The limit of detection was found to be 101.1 ppm.^81^ The information about the response time and the concentration of pesticides at which the sensors responded to all the four sensors is given in Table 6.

**Table 6:** Information about the response time and minimum concentration of three pesticides for the four sensors under test. Data are extracted from Fig. 11–14

Sensor composed of	Response to pesticide P_1_		Response to pesticide P_2_		Response to pesticide P_3_	
	Minimum concentration (v/v)	Response time (min)	Minimum concentration (v/v)	Response time (min)	Minimum concentration (v/v)	Response time (min)
Pure HCDA	0.3	1	0.24	1	0.3	10
HCDA/AgNP (5%)	0.18	1	0.18	2	0.24	8–10
HCDA/AgNP (10%)	0.06	Less than 1 min	0.06	Less than 1 min	0.3	5
HCDA/AgNP (15%)	0.12	1	0.12	2-3	0.18	10

**Fig. 15:**
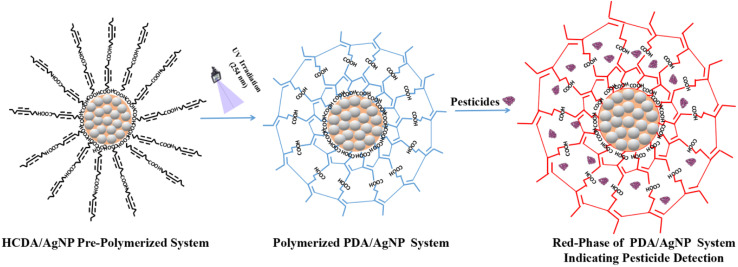
Schematic representation of the sensing mechanism.

In the PDA/AgNP system, AgNPs are negatively charged and have high affinity towards the carboxylate group of PDA. In addition, there exists the H-bond that could interact with the carboxylate group of PDA head part *via* ionic interactions. The intermolecular H-bond within the PDA structure may facilitate the adsorption of PDA on the surface of AgNP.^78^ Overall, sphere-like structures are formed in which AgNPs are surrounded by PDA [Fig. 15]. The formation of single bilayer structures of HCDA in the colloid has been reported.^46^ In the present case, when the PDA/AgNP system was exposed to pesticides, the pesticide molecules may be incorporated in between the ordered layers of PDA within the PDA/AgNP nanohybrid complex^26,78^ [Fig. 15]. This may result in the straightening of hydrocarbon chains of PDA, leading to the attainment of red phase at a faster rate than that in the case of pure PDA. It has been reported that such straightening of PDA chains influence the attainment of red phase.^82,83^

The present sensor responded to three pesticides, namely, P_1_, P_2_ and P_3_. In order to check the selectivity of the designed sensor, the sensors have been tested with other two pesticides, namely, urea and glyphosate. However, no significant response was observed [Fig. S6 of the ESI†].

The observed selectivity in sensing performance can be attributed to the molecular characteristics of the pesticides. The tested pesticides—P_1_, P_2_ and P_3_—contain hydrophobic moieties and specific functional groups (such as halogens and aromatic rings) that allow their incorporation within the PDA layers, leading to a visible color change. However, urea and glyphosate do not induce a response, probably due to their strongly hydrophilic nature and different charge distributions, which hinder their effective incorporation into the PDA/AgNP complex. Unlike the previously tested pesticides, these molecules may not interact efficiently with the PDA matrix or disrupt its ordered structure, thereby preventing the colorimetric transition. The presence of different ionic or highly polar functional groups in these molecules may also play a role in their lack of interaction with the AgNP-PDA system.

This selective response suggests that both the structural compatibility of the three pesticides under investigation with the PDA layers and their ability to interact with the AgNP-PDA matrix play crucial roles in governing the sensing mechanism.

## 4 Conclusion

In conclusion, we have successfully fabricated a user-friendly and economic paper-based colorimetric pesticide sensing platform using green-synthesized AgNPs and PDA. AgNPs have been synthesized using *A. heterophylla* leaf extract. Photopolymerization and blue-to-red phase conversion of PDA have been optimized. It has been observed that AgNPs influence the phase change of HCDA and HCDA/AgNP sensors, with 10% of AgNPs as the optimum for maximum blue-to-red phase conversion. Using HCDA/AgNP, a paper-based colorimetric pesticide sensor has been designed. The sensor has been tested with three locally available and frequently used pesticides, and satisfactory results were obtained. The proposed sensor is portable and economic. It is just like litmus paper, where colour change gives the idea about sensing. A MATLAB-based system has been used to analyze the RGB values, and the NSI was calculated to quantify the sensing process. All the designed sensors have been tested with the real samples collected from an agricultural field, and satisfactory results were observed. As a whole, we have demonstrated a HCDA/AgNP-based paper sensor which has the potential to overcome the limitation of sophisticated costly pesticide sensing methods and offer a cost-effective, portable and user-friendly pesticide detection system.

## Data availability

The data will be made available upon reasonable request.

## Author contributions

S. A. H.: supervision, conceptualization, investigation, funding acquisition, resources, validation, writing – review & editing; S. H.: conceptualization, data curation, methodology, formal analysis, visualization, writing – original draft, writing – review & editing; D. S.: data curation, methodology; S. M.: data curation, writing – review & editing; H. A.: data curation; D. B.: formal analysis; K. A. A.: formal analysis, funding acquisition; A. N. A.: formal analysis, funding acquisition.

## Conflicts of interest

The authors declare no conflict of interest.

## Supplementary Material

RA-015-D5RA01243K-s001

## References

[cit1] Goel A., Aggarwal P. (2007). Pesticide poisoning. Natl. Med. J. India.

[cit2] Zhang L., Yin X., Yang H., Wen H., Han S., Pan X., Li H., Peng D. (2023). Foods.

[cit3] Rodrigues A. C., Barbieri M. V., Febbraio F. (2022). EFSA.

[cit4] Majumder S., Deb S., Hussain S., Dey D., Bhattacharjee D., Alodhayb A. N., Hussain S., Hussain S. A. (2025). Sci. Rep..

[cit5] Yang Y., Yu H., Yang Y., Zhang J., Yin J., Shao B. (2021). ACS Agric. Sci. Technol..

[cit6] Stachniuk A., Fornal E. (2016). Food Anal. Methods.

[cit7] Baskaran S., Kookana R. S., Naidu R. (1997). J. Chromatogr. A.

[cit8] Lu D., Shao G., Du D., Wang J., Wang L., Wang W., Lin Y. (2011). Lab Chip.

[cit9] Dissanayake N. M., Arachchilage J. S., Samuels T. A., Obare S. O. (2019). Talanta.

[cit10] Tseng M.-H., Hu C.-C., Chiu T.-C. (2019). Dyes Pigm..

[cit11] Zhang Y., Bromberg L., Lin Z., Brown P., Van Voorhis T., Hatton T. A. (2018). J. Colloid Interface Sci..

[cit12] Alam A. K. M. M., Jenks D., Kraus G. A., Xiang C. (2021). Nanomaterials.

[cit13] Hussain S., Deb R., Suklabaidya S., Bhattacharjee D., Arshad Hussain S. (2022). Mater. Today: Proc..

[cit14] Wen J. T., Roper J. M., Tsutsui H. (2018). Ind. Eng. Chem. Res..

[cit15] Sergi R., Brugnoli B., Sturabotti E., Piozzi A., Galantini L., Taresco V., Francolini I. (2023). Macro Chem. Phys..

[cit16] Motaghedi F., Rose L., Sur A. K., Garg G., Nyayachavadi A., Ahamed M. J., Carmichael T. B., Rondeau-Gagné S. (2024). Adv. Mater. Technol..

[cit17] Chen W., Hazoor S., Madigan R., Adones A. A., Chintapula U. K., Nguyen K. T., Tang L., Foss F. W., Dong H. (2022). Mater. Today Adv..

[cit18] Finney T. J., Frank S. L., Bull M. R., Guy R. D., Kuhl T. L. (2023). Adv. Mater. Inter..

[cit19] Hussain S., Majumder S., De U. C., Bhattacharjee D., Hussain S., Hussain S. A. (2024). Interactions.

[cit20] Chen S.-W., Chen X., Li Y., Yang Y., Dong Y., Guo J., Wang J. (2022). RSC Adv..

[cit21] Kim C., Hong C., Lee K. (2021). Biosens. Bioelectron..

[cit22] Hussain S., Majumder S., Malhotra A., Chauhan A., Bhattacharjee D., Hussain S. A. (2023). J. Mater. Sci..

[cit23] Kaewtong C., Wanno B., Rakrai W., Saenkham A., Sriphalang S., Pattavarakorn D., Tuntulani T., Pulpoka B. (2024). Environ. Technol..

[cit24] Huo J., Deng Q., Fan T., He G., Hu X., Hong X., Chen H., Luo S., Wang Z., Chen D. (2017). Polym. Chem..

[cit25] Yapor J. P., Alharby A., Gentry-Weeks C., Reynolds M. M., Alam A. K. M. M., Li Y. V. (2017). ACS Omega.

[cit26] Saenjaiban A., Singtisan T., Suppakul P., Jantanasakulwong K., Punyodom W., Rachtanapun P. (2020). Polymers.

[cit27] Zhou H. S., Wada T., Sasabe H., Komiyama H. (1996). Synth. Met..

[cit28] Su Y. (2006). React. Funct. Polym..

[cit29] Song W., Li Y., Geng L., Feng G., Ren J., Yu X. (2021). Mater. Des..

[cit30] Tobias A., Rooke W., Hanks T. W. (2019). Colloid Polym. Sci..

[cit31] Phonchai N., Khanantong C., Kielar F., Traiphol R., Traiphol N. (2020). Colloids Surf. A Physicochem. Eng. Asp..

[cit32] Park K. H., Oh S., Kim J., Yang S. Y., An B.-S., Hwang D. Y., Lee J. H., Kim H. S., Lee J., Seo S. (2020). J. Exp. Nanosci..

[cit33] Kim J., Moon B.-S., Hwang E., Shaban S., Lee W., Pyun D.-G., Lee D. H., Kim D.-H. (2021). Analyst.

[cit34] Lu S., Luo F., Duan X., Jia C., Han Y., Huang H. (2014). J. Appl. Polym. Sci..

[cit35] Ito-Washiyama W., Onodera T., Ageishi M., Sato R., Zhang B., Kato S., Masuhara A., Kasai H., Mamiya H., Jinnai H., Takeda Y., Oikawa H. (2022). J. Phys. Chem. C.

[cit36] Cui C., Kim S., Ahn D. J., Joo J., Lee G. S., Park D. H., Kim B.-H. (2018). Synth. Met..

[cit37] Liffmann R., Homberger M., Mennicken M., Karthäuser S., Simon U. (2015). RSC Adv..

[cit38] Won S. H., Sim S. J. (2012). Analyst.

[cit39] Chen X., Li L., Sun X., Liu Y., Luo B., Wang C., Bao Y., Xu H., Peng H. (2011). Angew. Chem., Int. Ed..

[cit40] Nopwinyuwong A., Boonsupthip W., Pechyen C., Suppakul P. (2013). Adv. Polym. Technol..

[cit41] Yokoyama T., Masuhara A., Onodera T., Kasai H., Oikawa H. (2011). J. Phys. Chem. C.

[cit42] Burris A. J., Cheng Q. (2021). Langmuir.

[cit43] Akiyama T., Masuhara A., Arakawa T., Munaoka T., Onodera T., Oikawa H., Yamada S. (2011). Jpn. J. Appl. Phys..

[cit44] Bhushan B., Kundu T., Singh B. P. (2014). Opt. Commun..

[cit45] Alloisio M., Zappia S., Demartini A., Petrillo G., Ottonelli M., Thea S., Dellepiane G., Muniz-Miranda M. (2014). Mater. Chem. Phys..

[cit46] Alloisio M., Zappia S., Demartini A., Espinoza M. I. M., Ottonelli M., Dellepiane G., Thea S., Cavalleri O., Rolandi R. (2015). Nano-Struct. Nano-Objects.

[cit47] Asif M., Yasmin R., Asif R., Ambreen A., Mustafa M., Umbreen S. (2022). Dose Response.

[cit48] Suklabaidya S., Chakraborty S., Sarkar S., Paul R., Banik H., Chakraborty A., Bhattacharjee D., Majumdar S., Hussain S. A. (2021). J. Phys. Chem. C.

[cit49] Anastassiades M., Lehotay S. J., Štajnbaher D., Schenck F. J. (2003). J. AOAC Int..

[cit50] Nguyen V. P., Le Trung H., Nguyen T. H., Hoang D., Tran T. H. (2021). J. Nanomater..

[cit51] Shankar T., Karthiga P., Swarnalatha K., Rajkumar K. (2017). Resource-Efficient Technologies.

[cit52] Dong X., Ji X., Jing J., Li M., Li J., Yang W. (2010). J. Phys. Chem. C.

[cit53] Sahin Yaglioglu A., Erenler R., Gecer E. N., Genc N. (2022). J. Inorg. Organomet. Polym..

[cit54] Huq Md. A. (2020). IJMS.

[cit55] Mohammadalinejhad S., Almasi H., Esmaiili M. (2019). Mater. Sci. Eng. C.

[cit56] Lomelí-Rosales D. A., Zamudio-Ojeda A., Reyes-Maldonado O. K., López-Reyes M. E., Basulto-Padilla G. C., Lopez-Naranjo E. J., Zuñiga-Mayo V. M., Velázquez-Juárez G. (2022). Molecules.

[cit57] Lavanya K., Kalaimurugan D., Shivakumar M. S., Venkatesan S. (2020). J. Clust. Sci..

[cit58] Arroyo G., Angulo Y., Debut A., Cumbal L. H. (2021). Catalysts.

[cit59] Chinnasamy G., Chandrasekharan S., Bhatnagar S. (2019). Indian J. Nephrol..

[cit60] Akhatova F., Konnova S., Kryuchkova M., Batasheva S., Mazurova K., Vikulina A., Volodkin D., Rozhina E. (2023). IJMS.

[cit61] Rastogi L., Arunachalam J. (2011). Mater. Chem. Phys..

[cit62] Selvaraj R., Nagendran V., Varadavenkatesan T., Goveas L. C., Vinayagam R. (2024). Chem. Eng. Res. Des..

[cit63] Cakić M., Glišić S., Cvetković D., Cvetinov M., Stanojević L., Danilović B., Cakić K. (2018). Colloid J..

[cit64] Firdaus M. L., Fitriani I., Wyantuti S., Hartati Y. W., Khaydarov R., Mcalister J. A., Obata H., Gamo T. (2017). Anal. Sci..

[cit65] Akhter M. S., Rahman M. A., Ripon R. K., Mubarak M., Akter M., Mahbub S., Al Mamun F., Sikder M. T. (2024). Heliyon.

[cit66] Masum M. M. I., Siddiqa M. M., Ali K. A., Zhang Y., Abdallah Y., Ibrahim E., Qiu W., Yan C., Li B. (2019). Front. Microbiol..

[cit67] Peulen T.-O., Wilkinson K. J. (2011). Environ. Sci. Technol..

[cit68] Elamawi R. M., Al-Harbi R. E., Hendi A. A. (2018). Egypt J. Biol. Pest Control.

[cit69] Nahar K., Aziz S., Bashar M., Haque Md. A., Al-Reza S. Md. (2020). Int. J. Nano Dimens..

[cit70] Peloquin D. M., Baumann E. J., Luxton T. P. (2020). Chemosphere.

[cit71] Raj S., Chand Mali S., Trivedi R. (2018). Biochem. Biophys. Res. Commun..

[cit72] Suklabaidya S., Chakraborty S., Sarkar S., Paul R., Banik H., Chakraborty A., Bhattacharjee D., Majumdar S., Hussain S. A. (2021). J. Phys. Chem. C.

[cit73] Chowdhury S., Yusof F., Sulaiman N., Faruck M. O. (2017). SSP.

[cit74] Seki T., Tanaka K., Ichimura K. (1998). Polym. J..

[cit75] Suklabaidya S., Hussain S., Sarkar S., Majumdar S., Bhattacharjee D., Hussain S. A. (2024). Interactions.

[cit76] Beliktay G., Shaikh T., Koca E., Cingil H. E. (2023). ACS Omega.

[cit77] Wenzel M., Atkinson G. H. (1989). J. Am. Chem. Soc..

[cit78] Chen X. Y., Li J. R., Pang S. J., Shen A. L., Jiang L. (1999). Surf. Sci..

[cit79] Champaiboon T., Tumcharern G., Potisatityuenyong A., Wacharasindhu S., Sukwattanasinitt M. (2009). Sensor. Actuator. B Chem..

[cit80] Tu M.-C., Cheema J. A., Yildiz U. H., Palaniappan A., Liedberg B. (2017). J. Mater. Chem. C.

[cit81] Shrivastava A., Gupta V. (2011). Chron. Young Sci..

[cit82] Lifshitz Y., Golan Y., Konovalov O., Berman A. (2009). Langmuir.

[cit83] Deb P., Yuan Z., Ramsey L., Hanks T. W. (2007). Macromolecules.

