# Supplementary material for: Green-synthesized silver nanoparticle-modulated polydiacetylene-based colorimetric pesticide sensor
Source: RSC Adv. 2025 Jun 2;15(23):18372–91. doi: 10.1039/d5ra01243k (PMC12128055; doi:10.1039/d5ra01243k)
Supplement: RA-015-D5RA01243K-s001 [file RA-015-D5RA01243K-s001.pdf]

Supplementary Information (SI) for RSC Advances.
This journal is © The Royal Society of Chemistry 2025

Supplementary Information

Greenly Synthesized Silver Nanoparticle Modulated Polydiacetylene Based Colorimetric Pesticide Sensor

*Shazidul Hussain[1], Dipan Sarma[2], Sangita Majumder[1], Debajyoti Bhattacharjee[1], Khuloud A. Alibrahim[3], Abdullah N. Alodhayb[4], Hemant Agarwal[5] and Syed Arshad Hussain[*1]*

[1]Thin Film and Nanoscience Laboratory, Department of Physics, Tripura University, Suryamaninagar, Agartala 799022, West Tripura, Tripura, India.

[2]Department of Botany, Tripura University, Suryamaninagar, Agartala 799022, West Tripura, Tripura, India.

[3]Department of Chemistry, College of Science, Princess Nourah bint Abdulrahman University, Riyadh 11671, Saudi Arabia.

[4]Research Chair for Tribology, Surface, and Interface Sciences, Department of Physics and Astronomy, College of Science, King Saud University, Riyadh, Saudi Arabia.

[5]Barcelona School of Telecommunications Engineering, Universitat Politècnica de Catalunya, Barcelona Tech, Carrer de Jordi Girona, 1-3, Les Corts, 08034 Barcelona, Spain.

[*] Corresponding author

Email: sa_h153@hotmail.com, sahussain@tripurauniv.ac.in

Ph/:+91940212250 (M), Fax: +913812374802 (O)

Orchid id: 0000-0002-3298-6260

(a)

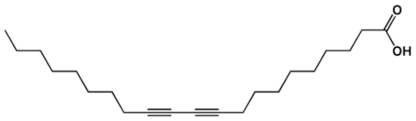

(b)

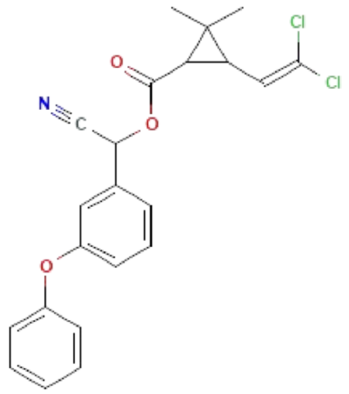

(c)

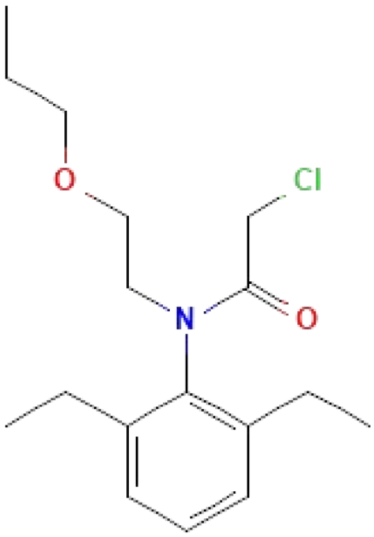

(d)

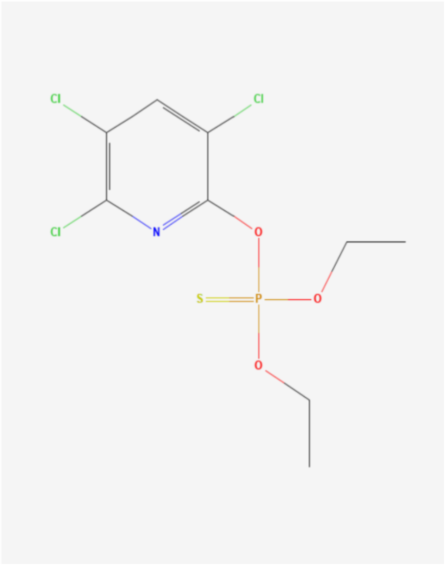

Fig. S1: Molecular structures of (a) 10,12- Henicosadiynoic acid (b) Cypermethrin (IUPAC Name: cyano-(3-phenoxyphenyl)methyl] 3-(2,2 dichloroethenyl)-2,2-dimethylcyclopropane-1-carboxylate) (P_1) (c) Pretilachor (IUPAC Name: 2-chloro-*N*-(2,6-diethylphenyl)-*N*-(2-propoxyethyl)acetamide) (P_2) and Chlorpyriphos (IUPAC Name: O,O-diethyl O-(3,5,6-trichloro-2-pyridyl) phosphorothioate (P_3)

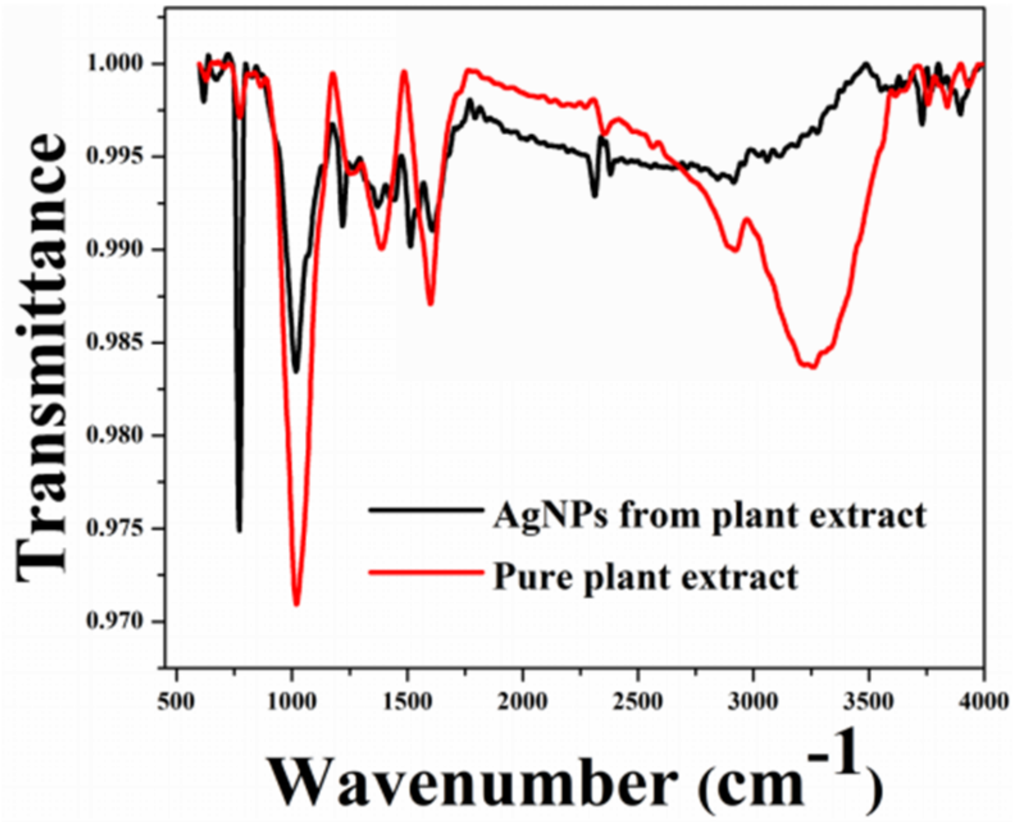

Fig. S2: FTIR spectra of *A. heterophylla* leaves extract and bio synthesized AgNPs

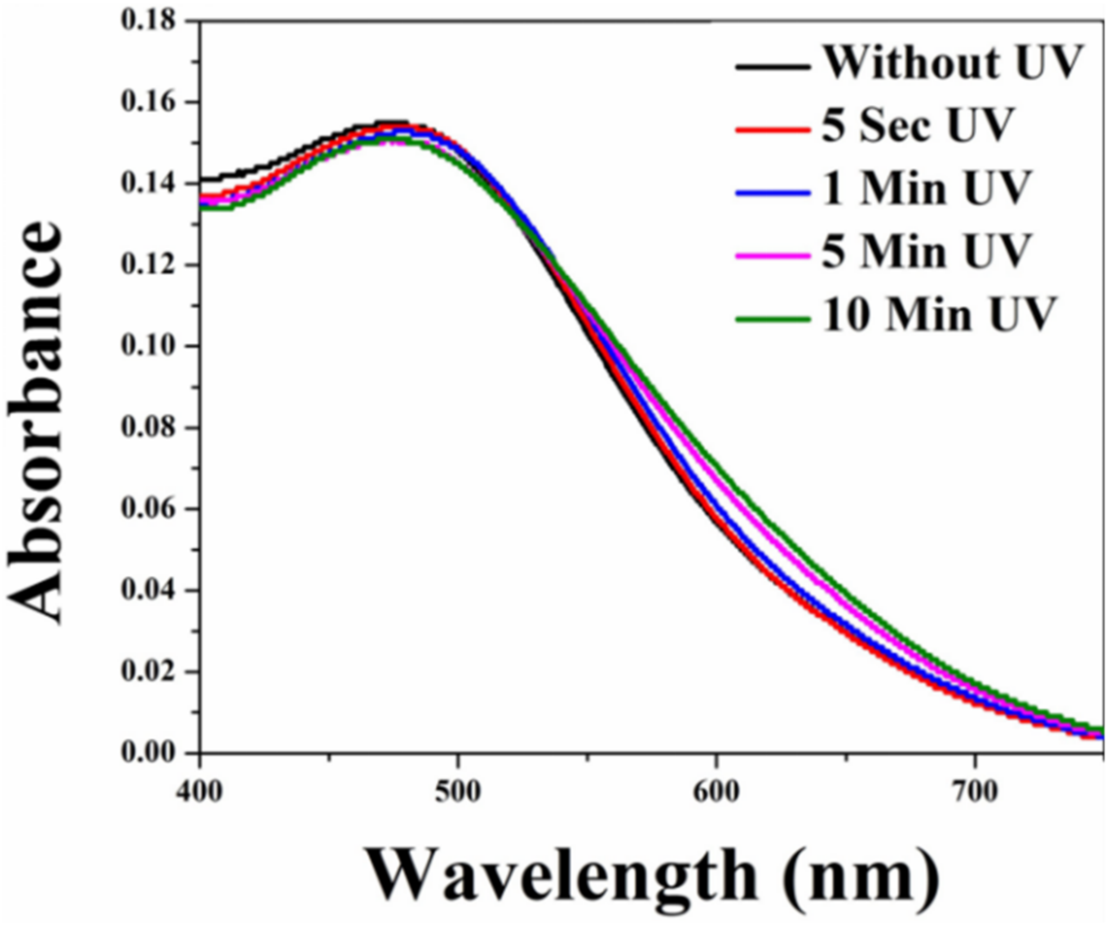

Fig.S3: UV-Vis absorption spectra of pure AgNP self standing film upon UV-irradiation for various exposure time

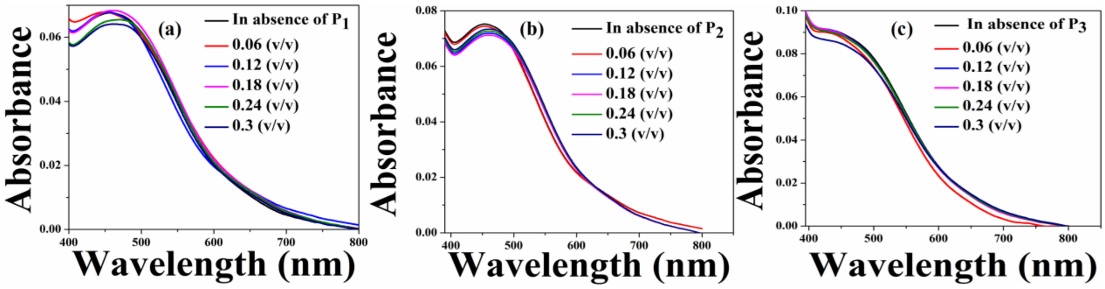

Fig. S4: UV-Vis absorption spectra of AgNPs in the presence of (a) P_1, (b) P_2 and (c) P_3 varying different concentration

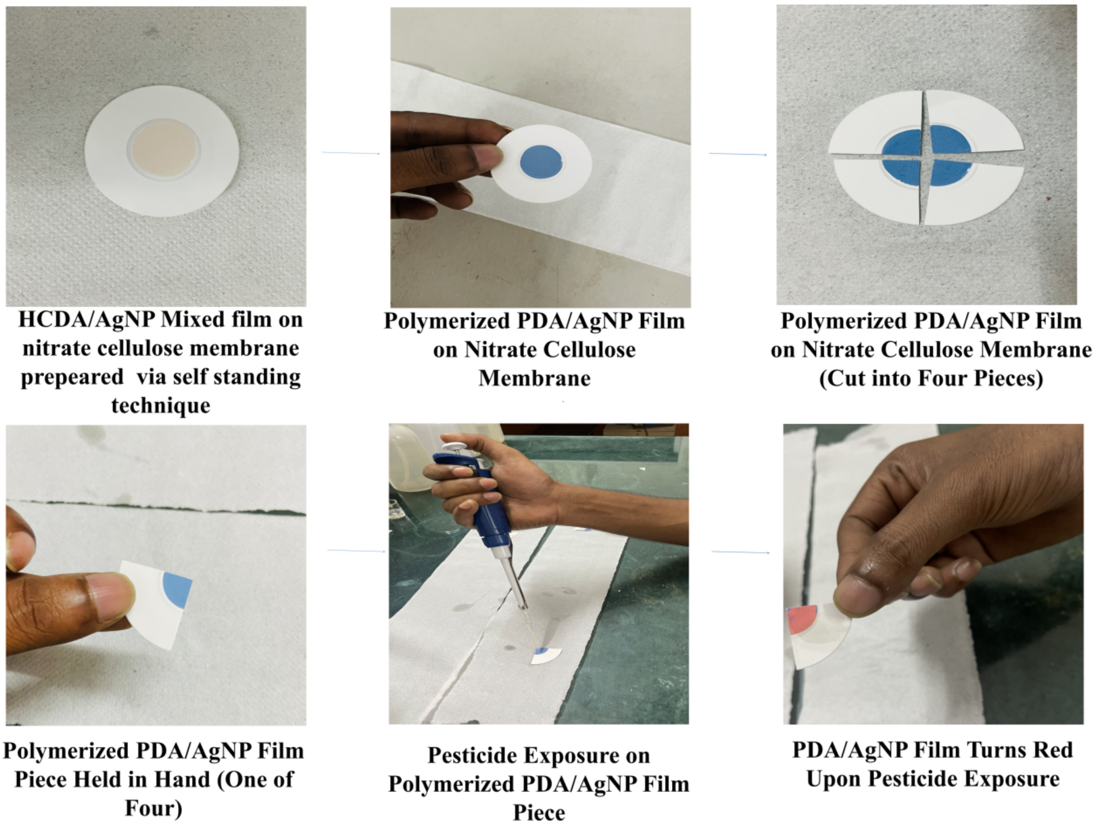

Fig. S5: Demonstration of Sensor Application

The PDA/AgNP-based colorimetric sensor was fabricated using a self-standing technique on a nitrate cellulose membrane. Upon polymerization under UV 254 nm, the film turned into its blue phase. To facilitate practical application, the film was cut into four equal pieces for testing. During the sensing process, one of the film pieces was exposed to a pesticide solution. Upon exposure, the film has changed its color from blue to red, indicating the presence of pesticides. This naked eye visible change is due to the interaction between the pesticide molecules and the PDA/AgNP composite, which disturbs the conjugated polymer backbone, leading to a chromatic change.

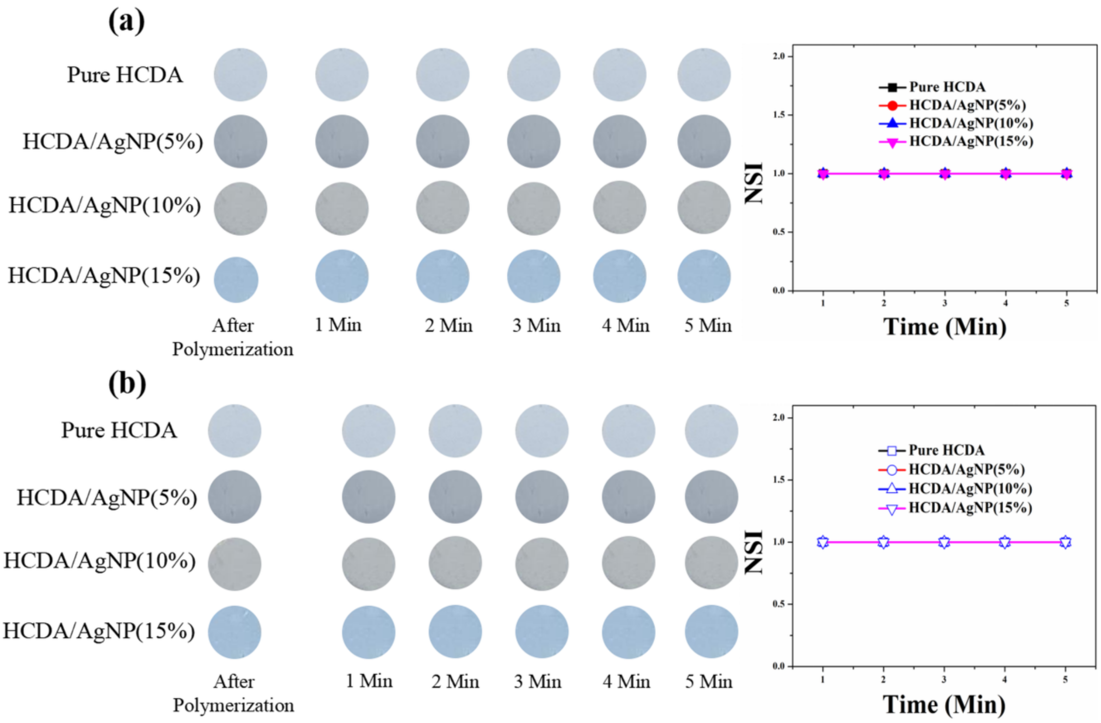

Fig. S6: Time dependant colorimetric response of pure HCDA and HCDA/AgNP mixed paper based sensor upon exposure of (a) Urea and (b) Glyphosate at a concentration of 1000 ppm. The respective NSI values are presented alongside each set of colorimetric transitions, demonstrating the sensor's response across time intervals.
